# Grammars of yoga: an exploratory text analytics approach to classical, pedagogical, scholarly, and scientific corpuses of yoga

**DOI:** 10.3389/fpsyg.2026.1908435

**Published:** 2026-10-26

**Authors:** Sabbi Lall, Jinjie Liu, Swayam Bagaria

**Affiliations:** 1 Harvard Divinity School, Harvard University, Cambridge, MA, United States; 2 The Institute for Quantitative Social Science, Harvard University, Cambridge, MA, United States

**Keywords:** corpus, grammar, indigenous, practice, tradition, yoga

## Abstract

**Introduction:**

Originating in Indian contemplative and musical traditions, yoga has transformed into a globalized set of psychophysiological and psychospiritual practices. Its worldwide spread has generated diverse formats and frameworks within which it is understood, including classical texts, modern practice manuals, medical research developing yoga-based interventions, and the academic field of yoga studies. However, it is unclear to what degree, if at all, this multiplication of formats and frameworks of yoga leads to a convergence in the articulation of the practices and aims of yoga between these different epistemic standpoints of conceptualizing yoga. Our paper addresses this question by outlining an inductive and computational approach to provide an empirical map of the grammars of yoga of these four corpuses of yoga.

**Methods:**

Two hundred text extracts (50 per source) were compiled from translated classical scriptures, recent biomedical research articles, modern yoga practice manuals, and academic yoga studies publications. Each extract comprised a locatable “yoga is…”-style statement plus surrounding context for natural language processing. We cleaned and tokenized the data, then applied both an unsupervised LDAMallet topic model and a semi-supervised GuidedLDA model seeded with anchor terms, allowing the topics to emerge inductively from the corpus rather than from predetermined categories. The number of topics was selected using coherence-score sensitivity analysis. We visualized results with pyLDAvis and t-SNE and tested them statistically using chi-squared analysis and bias-reduced multinomial logistic regression to assess associations between topics and source types.

**Results:**

Text analytics produced four well-separated, stable topics in our sample of 200 texts, each dominated by vocabulary with a suggested thematic orientation: (1) interventionist/outcome-focused, (2) experiential/phenomenological, (3) genealogical/historical, and (4) soteriological/contemplative. Statistical testing confirmed strong, non-random associations between each topic and a specific corpus, with predicted assignment probabilities exceeding 0.7, and named and weighted the specific terms distinguishing each grammar.

**Discussion:**

The presence of stable semantic signatures revealed signals of a latent sufficiently distinctive grammar for each of the four corpuses of yoga. The strong association between the source type and grammar showed that despite the vast heterogeneity across the corpuses of yoga, there are still four identifiable semantic patterns in which its practices and aims are framed. We discuss the stability of distribution as the result of the possible canalizing effects that the institutional location of the yoga practice has on the way in which its practices and goals are framed, even as their might be a more open-ended borrowing of themes and ideas at the level of actual practice. In providing a preliminary empirical validation of this pattern, our paper provides an avenue for future work that aims to create a dialogically plural and mutually enriching field of yoga studies and practice across its different institutional sites.

## 1 Introduction

Originating in Indian classical traditions of contemplative and mystical philosophy and practice, yoga is now recognized as a diverse set of psychophysiological and psychospiritual techniques that are practiced worldwide (Govindaraj et al., 2016; Schmalzl et al., 2021). This global dissemination of yoga has resulted in a multiplication of the formats and frameworks within which it is practiced and understood, far beyond those specified in the classical sources. In addition to the classical sources of yoga, there now exists a corpus of modern yoga practice manuals that frame the practice of yoga using a mix of postural, meditative, and spiritual values and aims; a field of medical and scientific research that aims to create specific yoga-based interventions and protocols by testing the benefits of yoga as a mind–body practice and alternative medicine; and, finally, there is an academic discipline, yoga studies, that provides a more historical and anthropological overview of the multiple lineages through which the philosophies and practices of yoga have been transmitted over time. However, it is unclear to what degree, if at all, this multiplication of formats and frameworks of yoga actually leads to a convergence in the articulation of the practices and aims of yoga between these different epistemic standpoints of conceptualizing yoga. Our paper addresses this question by outlining an inductive and computational approach to provide an empirical map of the grammars of yoga of these four corpuses of yoga.

Crucially, the goal of mapping these grammars is not to collapse them into a single, unified account or ‘true’ language of yoga. We agree with the current scholarly consensus in yoga studies that there is no unifying standpoint from which to understand the diverse practices and aims of yoga. This impulse is captured well in the description that the scholar Andrea Jain provides of the field of yoga practice:

“a collection of complex data made up of a congeries of figures, institutions, ideas, and practical paths involving mental or physical techniques – most commonly meditative, breathing, or postural exercises. It is believed to resolve the problem of suffering and to improve health, both defined in modern terms. Postural yoga often betrays a desire to repair what is perceived as an imbalance of ‘body-mind-soul.’ Finally, it is tied to mythologies about the historical transmission of yogic knowledge, accumulating around a transnational community that has engaged in and transmitted what participants call yoga – and sometimes refuse to call yoga – since the twentieth century.” (Jain, 2014).

Scholars such as Foxen and Kuberry (2021) go even further by arguing that not only is there no single way to organize the field of yoga, given its diversity across scriptures and modern practice, but, in fact, the only way out is to study particular traditions or practices of yoga and the network of influences and contexts within which it is embodied (Jain, 2014; Newcombe, 2018). They make their case by pointing to the heterogeneity of the classical archive of yoga itself and its diverse and multifarious lines of transmission and development (Mallinson and Singleton, 2017). Even the word “yoga” has a broad range of meanings in the Sanskrit lexicon (White, 2014), is used to nominally describe very distinct practices and soteriological ends in Hindu and non-Hindu traditions. Even a central text of yoga, such as *The Yogasūtras of Patañjali*, has been described by scholars as a “partisan text, representing an early Brahmanical appropriation of extra-Vedic, *śramaṇa* techniques of yoga, such as those of early Buddhism” (Mallinson and Singleton, 2017). In the context of such a diverse classical archive, the most valid approach would be to study the specific sectarian and textual lineage through which a practice of yoga has developed. Furthermore, these scholars see the necessity of a highly contextual approach even more when one considers the hybrid and experimental influences that modern yoga practitioners have employed to define their own practice and understanding of yoga. For example, Tirumalai Krishnamacharya, the pioneer of modern hatha yoga in India, created a blend of yogic techniques inspired by mixing postures from classical hatha yoga texts with elements from European calisthenics and the dynamics of wrestling traditions of South India, where he lived and worked (Singleton, 2010). Contrast this with the figure of Swami Kuvalyananda, the founder of Kaivalyadham school of yoga, who articulated the aims of yoga by framing the fortifying effects it has on the body in the language of contemporary scientific research such as ‘increasing the body’s threshold of reactivity’ (Kuvalayananda, 1963).

Given this heterogeneity, it is interesting to ask if there is an appropriate classification schema to organize this vast field of yoga practice across these diverse forms and formats. Yoga studies scholar De Michelis (2007) has provided one such top-down typology. She classifies modern yoga into postural, meditative, psychosomatic, denominational, and what she calls Neo-Hindu varieties. Her approach to classification is based on a reading of a selection of significant figures in the history of yoga and how their conceptual pivots allowed the field of yoga practice to multiply and proliferate. Singleton (2010) further expands upon De Michelis (2007) classification by documenting the historical importance of exercise culture and harmonial gymnastics for ‘creolizing’ yoga and allowing it to be framed as a practice to support physical health. While De Michelis (2007) relies on a top-down method and pre-determined categories of classification to typologize the corpus of modern yoga, our paper outlines a bottom-up, inductive, and computational approach to explore if there are any signals of an organizing schema that might emerge when we look at a broad genre of yoga texts.

In order to conduct this analysis, we apply topic modeling to extract a dataset of 200 definitions of yoga equally spread across four different corpora: (1) texts from the classical indigenous tradition on yoga, (2) yoga practice manuals across different lineages, (3) yoga used in recent scientific and medical literature on yoga, and (4) definitions of yoga provided in modern scholarly accounts of yoga. We specifically extract definitions from each text because of the framing effect that definitions have on the representation of the means and ends of yoga practice for that given text. In order not to predetermine the association between the topics and the source type, we removed all source labels and any other indication of the specific corpus from which the definition was extracted. Our analysis returned four distinct topics with a strong association between the source type and topic. We conducted a sensitivity analysis to confirm that four topics were the ideal number of topics to capture the distribution of topics across the entire dataset. While there were some atypical texts for which the topic did not match with the source type, each of the topics provided a stable semantic signature that was shared across the texts from the specific corpus with which it had a strong association. While this is not completely surprising for the biomedical corpus given the necessity of creating a standardized yoga protocol with very specific articulations about the aims of yoga, this was surprising for the other three corpuses given that each of the corpus contained a diverse array of texts that cut across timelines and regions. Our paper not only shows that the corpuses differed from each other in the way they conceptualize the practice and aims of yoga, it also provides an overview of the topics for each corpus, with each of the terms in the four topics named and weighted.

Our paper interprets this stable semantic signature for all four of the topics as signals of a latent grammar in each corpus. We use ‘grammar’ as an interpretive descriptor rather than as a direct synonym for the topics that we have identified through topic modeling. Conceptually, we use grammar in a Wittgensteinian sense (Wittgenstein, 1958) to refer to the linguistic field within which these different corpuses of yoga order their own experience of yoga and distinguish between terms and concepts that make sense to use in that context of yoga from those that do not. For Wittgenstein, understanding the grammar of a concept, or the latent network of other concepts within which that concept is located, is important to understanding the possible meanings the concept can take.

Although, for Wittgenstein, it was necessary to look beyond the linguistic network of concepts and understand the forms of life and contexts of use within which the concept is expressed, the semantic neighborhood of concepts still carried some of the traces of the forms of life within which it was concretized. Instead of equating our topic modeling results as direct suggestions of the presence of different grammars of yoga across varying corpuses, we consider our paper as contributing to the direction of future research on the different grammars of yoga. Such a disaggregation of the multiple grammars of yoga is important for understanding the endogenous ways in which the multiple corpuses of yoga might emphasize the mental, contemplative, and spiritual aspects of the practices and goals of yoga.

## 2 Materials and methods

### 2.1 Curation of definitions from distinct source types

We conducted literature analyses to aggregate definitions from four major corpora/source types related to yoga: classical scriptures, scientific studies examining the psychological and biomedical outcomes of yoga, analyses of yoga in humanistic scholarship, and modern yoga practice manuals from Indian and non-Indian teachers. We identified 50 examples from each source type, as described below. Figure 1 summarizes the overall flow of the study. In each case, we used a loose criterion to identify the primary definition of yoga (by locating the phrase “yoga is…” or a description of what yoga is) in that text and extracted that definition along with additional surrounding text (approximately 200 words) to create a robust pipeline for the topic modeling analysis. Since we extracted the definition of yoga for their framing effects, we also checked to see that each definition included a description of the ‘means’ or the suggested practice of yoga as well as some description of the ‘ends’ component or the aims of yoga. Citation markers were manually removed, and a final version of each definition, along with the surrounding context and the requisite metadata, was compiled (Supplementary Tables 1A,B).

**Figure 1:**
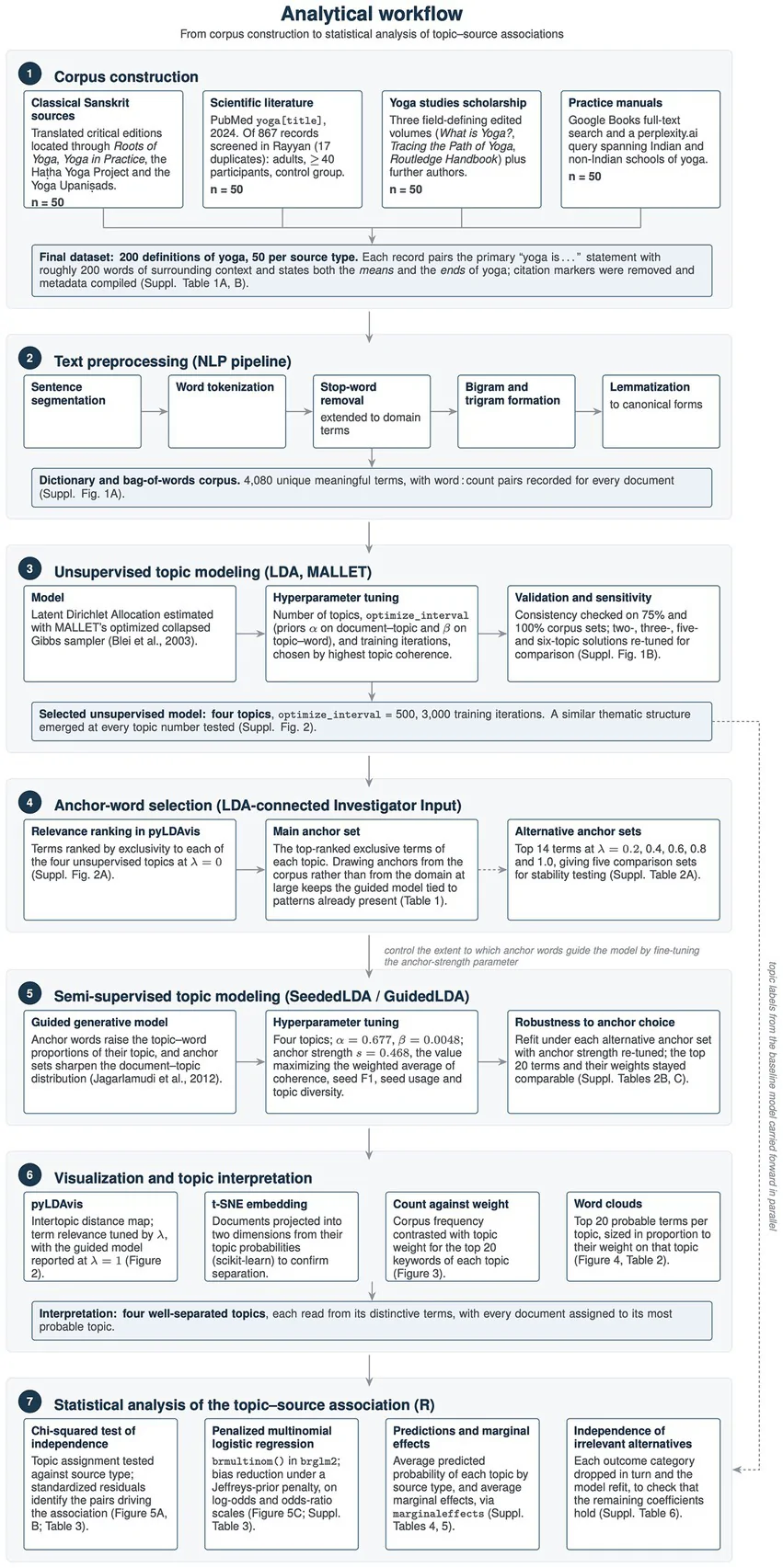
Steps in the overall analysis in this paper. The flow chart summarizes the analytic steps from corpus construction to statistical analysis of topic–source associations.

#### 2.1.1 Text extraction from scientific papers

We identified scientific papers by searching the predominantly biomedical database PubMed for “yoga [title],” and delimiting the search to the date range: 01-01-2024 to 01-01-2025. We limited the date range to obtain a recent snapshot of scientific research papers and reviews that discuss yoga. The resulting 867 articles were uploaded to rayyan.ai for sorting, with 17 identified as duplicates. Articles were included if yoga was in the title, and a definition of yoga was discussed in the Introduction. We extracted a yoga definition with context from the most recent 50 articles that examined populations aged 18 or older and demonstrated robust methodology: quantitative research articles were included if they examined 40 or more participants and had a control group. Two duplicated articles made it through the curation process (* in Supplementary Table 1A). Since the text duplication is exact, it should not change the outcome. The resulting articles came from a broad range of countries (Australia, Brazil, China, Germany, Greece, Hong Kong, India, Italy, Norway, Sweden, Turkey, UAE, UK, and the US; Supplementary Table 1B). By focusing on robust studies, we aimed to identify analyses that were most likely to include well-considered definitions of yoga.

#### 2.1.2 Text extraction from translations of Sanskrit sources

The sources for the classical, specifically Sanskrit, corpus, were chosen by selecting translated classical sources that were included in the collections *the Roots of Yoga* (Mallinson and Singleton, 2017) and *Yoga in Practice* (White, 2012). Both of these texts provide the most comprehensive and reliable collection of extracts and materials from the multiple source traditions of yoga. We also supplemented this by including texts from the Hatha Yoga Project^1^ that provided documentation of the latest source material for that particular tradition, as well as texts that were not present in the three sources above, such as the *Yoga Upaniṣads.* In each case, we first tried to use the critical editions of a text. If such editions did not exist, we used the best available English translations (cited in Supplementary Table 1A) to extract definitional text surrounding a “yoga is” style phrase, along with the appropriate context for those descriptions. Additionally, since the *Bhagavad Gītā* and the *Ṛg Veda* contain multiple distinct discussions of yoga, we included two distinct sections and, thus, two different sets of definitions of yoga from these important Sanskrit texts.

#### 2.1.3 Text extraction from yoga practice manuals

We searched Google Books for the term “yoga manual,” focusing on books with full text available to us, resulting in 26 manuals by largely American and European authors. We then used a query to search the internet for yoga manuals that were distributed across branches of yoga and including Indian schools. Duplicates in the list were excluded, as were texts that fell into the premodern Sanskrit sources category. We also checked our lists against the lists available in contemporary yoga studies scholarship (Kumar et al., 2021) to add any important manuals we were missing from across the different schools of yoga including *haṭha, karma, jñāna, rāja* yoga, as well as Iyengar, Ashtanga, Viniyoga, Kundalini, Shivananda, Bihar, Yin, Anusara, Jivamukti, Kripalu, Integral, Modern, Tantra, Yoga Therapy, and Vinyasa yoga. We excluded sources in which the full text was unavailable. For each of these texts, we searched the full text to extract the most appropriate statement of the way in which that school of yoga defined yoga (Supplementary Table 1A).

#### 2.1.4 Text extraction from academic yoga studies scholarship

The sources for this corpus were chosen by first looking at signature and influential edited volumes and journal issues in the humanities and human sciences that convened the most prominent scholars of yoga to provide an overview of the field of yoga studies. Specifically, we first searched three influential edited volumes - *What is Yoga*?, *Tracing the Path of Yoga,* and *The Routledge Handbook of Yoga and Meditation Studies* (Foxen and Kuberry, 2021; Sarbacker, 2021; Newcombe and O’Brien-Kop, 2021). All of these volumes offer a diverse and current overview of the academic field of yoga studies, and include chapters that explicitly discuss what yoga is to a range of specific communities. We supplemented this list by including other authors known to SB and SL in the Western academy. One important note here is that our selection criteria was skewed towards picking those trained in the Western scholarship traditions on yoga studies. This was because this is the intellectual landscape that both SB and SL are most familiar with. One important future step would be to determine the appropriate selection criteria for including Indian and other non-Western scholars in yoga studies.

#### 2.1.5 Final dataset

Overall, the final dataset comprised 200 definitions of yoga, including the surrounding textual context, evenly drawn from four types of sources. These sources span the ancient to contemporary time periods and come from a range of geographies (Supplementary Table 1B). Notably, the ancient sources are largely from India and span the ancient to early modern time periods, while subsequent corpora sit in the modern and contemporary eras.

### 2.2 Topic modeling analysis

We used text analytics, focusing on Topic Modeling to isolate distinct topics from the yoga definitions dataset (Supplementary Table 1A).

#### 2.2.1 Data cleaning

Next, we prepared the datasets for analysis. We used a standard Natural Language Processing (NLP) pipeline to clean each document (extracted from the various sources as outlined above) and to form separate meaningful words. This process involved splitting the text into sentences, splitting sentences into words, removing stop words, and extending stop words (to remove domain-specific, high-frequency, non-informative words such as yoga itself, and thus improve keyword extraction), creating bigrams and trigrams, and lemmatizing. The dictionary and corpus needed for Topic Modeling were then created from these meaningful words. The corpus has 4,080 unique meaningful words. For each document (text extracted from the different source types), this gives all word:count pairs. The word counts remaining after this data-cleaning step are shown in Supplementary Figure 1A.

Next, in addition to the traditional unsupervised word frequency-based Latent Dirichlet Allocation (LDA) method (section 2.2.2 below), we used a semi-supervised approach by manually adding “anchor words”/seeds (section 2.2.3 below). Both methods sort the same 200 definitions into four topics using patterns of word co-occurrence. They differ in whether the researcher tells the model where to start looking. Unsupervised LDA starts with nothing. It treats each topic as a cluster of words that tend to appear together and finds the clustering that best accounts for the corpus. The model cannot tell a word that defines a theme from one that is merely common within it, so the leading terms of each topic were often generic and the topics hard to name. We therefore also fitted GuidedLDA. Here the researcher supplies a short list of anchor words for each topic before fitting, terms that should be characteristic of that topic if it is real. The model still learns from the corpus in the same way, but it begins with a pull towards those terms and treats definitions containing them as more likely to belong to that topic. Topics formed around the anchors rather than around the most frequent words, which yielded the sharper, more interpretable themes we report. A full description of the topic modeling can be found in Supplementary methods.

#### 2.2.2 Unsupervised modeling using the LDAMallet model

To analyze the datasets for shared terms, we first used Latent Dirichlet Allocation (LDA) to run unsupervised topic modeling on our dataset (Blei et al., 2003). We used the Mallet module, which allows LDA model estimation using an optimized version of collapsed Gibbs sampling from MALLET, an efficient method for document-topic hyperparameter optimization. We used hyperparameter tuning to decide the optimal number of topics using three hyperparameters: (1) number of topics; (2) optimization of alpha (prior belief on document-topic distribution) and beta (prior belief on topic-word distribution) using “optimize_interval,” and (3) iterations, which tunes the number of training iterations.

We used two validation sets to test the consistency of the results: 75% corpus and 100% corpus. The optimal combination of the three hyperparameters, based on the highest topic coherence score, was four topics, with an optimized interval of 500, along with 3,000 training iterations. Therefore, these were the hyperparameter values used in the final LDA Mallet Model.

We also conducted a brief sensitivity analysis to check whether a similar thematic structure arose under different parameter settings. We tuned the three hyperparameters simultaneously: (1) number of topics; (2) optimize_interval: this optimizes alpha (a prior belief on document-topic distribution) and beta (a prior belief on topic-word distribution) automatically; and (3) iterations: number of training iterations. In Supplementary Figure 1B, we provide the values of the hyperparameters that reached the highest topic coherence score for two, three, five, and six topics.

We then fitted the LDAMallet Model with these hyperparameter values and visualized the results using pyLDAvis interactive plots. This revealed similar thematic structures emerged with two, three, five, or six topics, and the 4-topic model is the best model. We provide more details about the sensitivity analysis in Supplementary Methods Section 1.3.9.

#### 2.2.3 Semi-supervised modeling using the SeededLDA/GuidedLDA model

Since the most probable terms for topics from the LDAMallet model were sometimes generic, we used the SeededLDA/GuidedLDA model (Jagarlamudi et al., 2012), a semi-supervised LDA extension, to generate four more coherent topics. This approach allowed us to supply anchor words representative of the corpus to guide the topic discovery process. To achieve this, we used the pyLDAvis tool to obtain the top relevant terms ranked by their exclusiveness to the four topics uncovered by the LDAMallet model (Supplementary Figure 2A). The anchor words used to guide the semi-supervised model are provided in Table 1. Two aspects of the LDA generative process were modified to infuse the topic model with the user’s domain knowledge: topic-word proportions were upgraded using anchor words, and anchor sets were used to improve the document-topic distribution.

**Table 1:** Anchor words used for semi-supervised analysis.

Topic	Anchor words
Topic 1	[‘health’, ‘intervention’, ‘stress’, ‘breathing’, ‘improve’, ‘effect’, ‘benefit’, ‘treatment’, ‘relaxation’, ‘psychological’, ‘mental’, ‘balance’, ‘outcome’]
Topic 2	[‘experience’, ‘union’, ‘consciousness’, ‘divine’, ‘realization’, ‘communion’, ‘universe’, ‘spirit’, ‘perfection’, ‘peace’, ‘state’, ‘harmony’, ‘power’]
Topic 3	[‘text’, ‘century’, ‘philosophical’, ‘vedic’, ‘hindu’, ‘history’, ‘theory’, ‘jain’, ‘tradition’, ‘framework’, ‘logic’, ‘doctrine’]
Topic 4	[‘free’, ‘sound’, ‘object’, ‘air’, ‘ātman’, ‘mantra’, ‘fire’, ‘pleasure’, ‘bond’, ‘praise’, ‘bliss’, ‘contemplation’]

Anchor words were selected by visualizing the results of unsupervised topic modeling using a pyLDAvis interactive plot with λ = 0 to focus on distinctive terms in each topic. While the aim was to guide modeling with user knowledge, we essentially picked the top-ranked terms.

While this approach is justified, it also introduces investigator influence. However, this influence was minimal due to our approach. First, the anchor words used in GuidedLDA were derived from the initial unsupervised model with setting λ = 0 rather than being selected arbitrarily from the domain. With setting λ = 0, terms are ranked by their exclusiveness/uniqueness to the topic. This selection approach keeps the guided analysis connected to the patterns already found in the corpus and makes those patterns measured more reliable and coherent. The similarity between the two models indicates that GuidedLDA sharpened a thematic structure visible in the initial analysis rather than arbitrarily deviating it.

Second, although GuidedLDA incorporates investigator knowledge through the selection of anchor words, we fine-tuned the anchor-strength parameter, which controls the extent to which the model is guided by the investigator-specified anchors relative to the underlying word distributions in the corpus. This data-driven approach thus balances the influence of these domain-informed priors against the topic structure/patterns emerging from the corpus, reducing the potential for investigator bias to dominate the resulting topic structure. Although fine-tuning cannot completely remove investigator influence, it is a methodological safeguard against investigator-driven topic formation.

We tested different sets of anchor words to evaluate the stability of the results. We selected the top 14 terms of each topic when setting λ = 0.2, 0.4, 0.6, 0.8, and 1. This formed 5 alternative sets of anchor words. The main set of anchor words was selected from the top-ranked terms when setting λ = 0. The main and alternative sets of anchor words are listed in Supplementary Table 2A. Comparing these sets of anchor words reveals that many different words show up when λ = 0.2 versus λ = 0. However, as λ increases, only new general terms are included, and general terms become higher ranked than topic-specific terms. Using these alternative sets, we tested whether the resulting topics are sensitive to the very different sets of anchor words and/or to similar sets of anchor words but with a different order of within-set words. As sets of anchor words changed, we re-tuned the anchor-strength hyperparameter to look for its optimal value to maximize the weighted average of specified evaluation metrics. These hyperparameter values are presented in Supplementary Table 2B. We found that the top 20 probable terms and their weights to each topic are similar across models with different sets of anchor words (namely models with different values of λ). This is shown in Supplementary Table 2C.

Based on our initial unsupervised modeling, we again tuned the model to four topics. We found that alpha = 0.677, beta = 0.0048 maximized the weighted average of topic coherence and topic diversity of the model. Given four topics and these alpha and beta values, a specific anchor strength was chosen (*s* = 0.468) that maximized the weighted average of topic coherence, seed score (f1), seed usage score, and topic diversity of the model. Interestingly, the resulting topics were similar to the unseeded model with respect to the characteristic terms that fell out; while the topics remained well-separated, the distribution of bubbles in the pyLDAvis tool was shifted relative to the unsupervised model (compare Supplementary Figure 2, which shows the unsupervised model, to Figure 2 which shows semi-supervised results).

**Figure 2:**
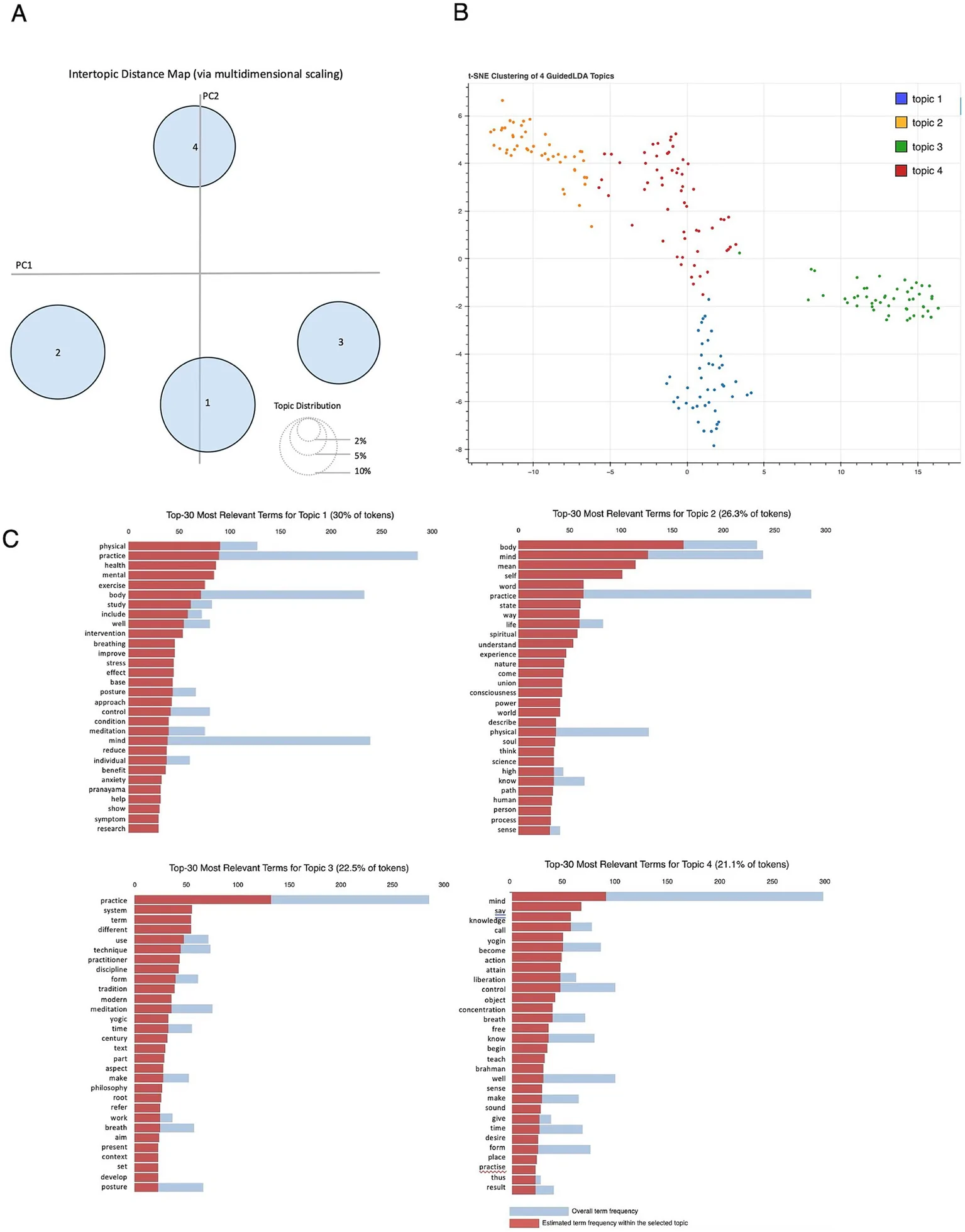
Well-separated topics emerge when the number of topics is limited to four. **(A)** Topic distributions in the semi-supervised model were generated using a pyLDAvis interactive plot. When the model is restricted to four topics, data visualization revealed four topics with big, non-overlapping bubbles scattered throughout the chart, indicating a good topic model. In the resulting Inter-topic Distance Map (left), circles represent different topics and indicate the distance between each topic. Similar topics appear closer and dissimilar topics farther apart. The relative size of a topic circle in the plot corresponds to the relative frequency of the topic in the corpus. The larger the bubble, the higher the percentage of documents in the corpus related to that topic. The image shows setting λ = 1, indicating the actual topic distribution. **(B)** t-SNE plot analyzing clustering of documents in 2D space in the semi-supervised LDA model. The plot confirms that documents (individual points) form four distinct topic clusters. Documents with low Euclidean distance between topic probabilities have similar t-SNE representations. Colors indicate documents assigned to the four topics during topic modeling, and the t-SNE algorithm confirms that documents cluster according to topic (blue = topic 1, yellow = topic 2, green = topic 3, red = topic 4). **(C)** The top 30 probable terms in the clusters in panel A, with λ = 1, i.e., indicating the actual topic distribution. Light blue bars indicate the overall term frequency while red bars show the estimated term frequency within the selected topic.

### 2.3 Visualization of resulting topics

#### 2.3.1 pyLDavis analysis

We used a pyLDAvis interactive plot to assess the robustness of topic models and select top keywords. We then examined relevance by tuning parameter λ, where a smaller λ gives higher weight to the term’s distinctiveness, while a larger λ corresponds to the probability of the term occurrence per topic. λ = 0 was used for initial topic interpretation and to select anchor words for the semi-supervised topic model. Final reporting (Figure 2 for the semi-supervised model and Supplementary Figure 2B for the unsupervised model) shows the top probable terms using λ = 1 to reflect the actual topic distribution in the unsupervised model and λ = 1 in the semi-supervised version.

#### 2.3.2 T-SNE clustering

We used t-SNE (t-distributed stochastic neighbor embedding) to cluster our topics and validate clear separation. We clustered the documents in 2D space using the t-SNE algorithm, with topic probabilities for each document as inputs. We used the Python scikit-learn manifold package to run the TSNE() function in Python.

#### 2.3.3 Word count and importance contrast of topic keywords for each topic

We contrasted word count and word importance for the top 20 keywords in each topic. Word count was calculated as the frequency of a given keyword in the whole corpus. Word importance was based on the weight of the word loading on a topic, i.e., the contribution/importance of the word to the topic (Figure 3).

**Figure 3:**
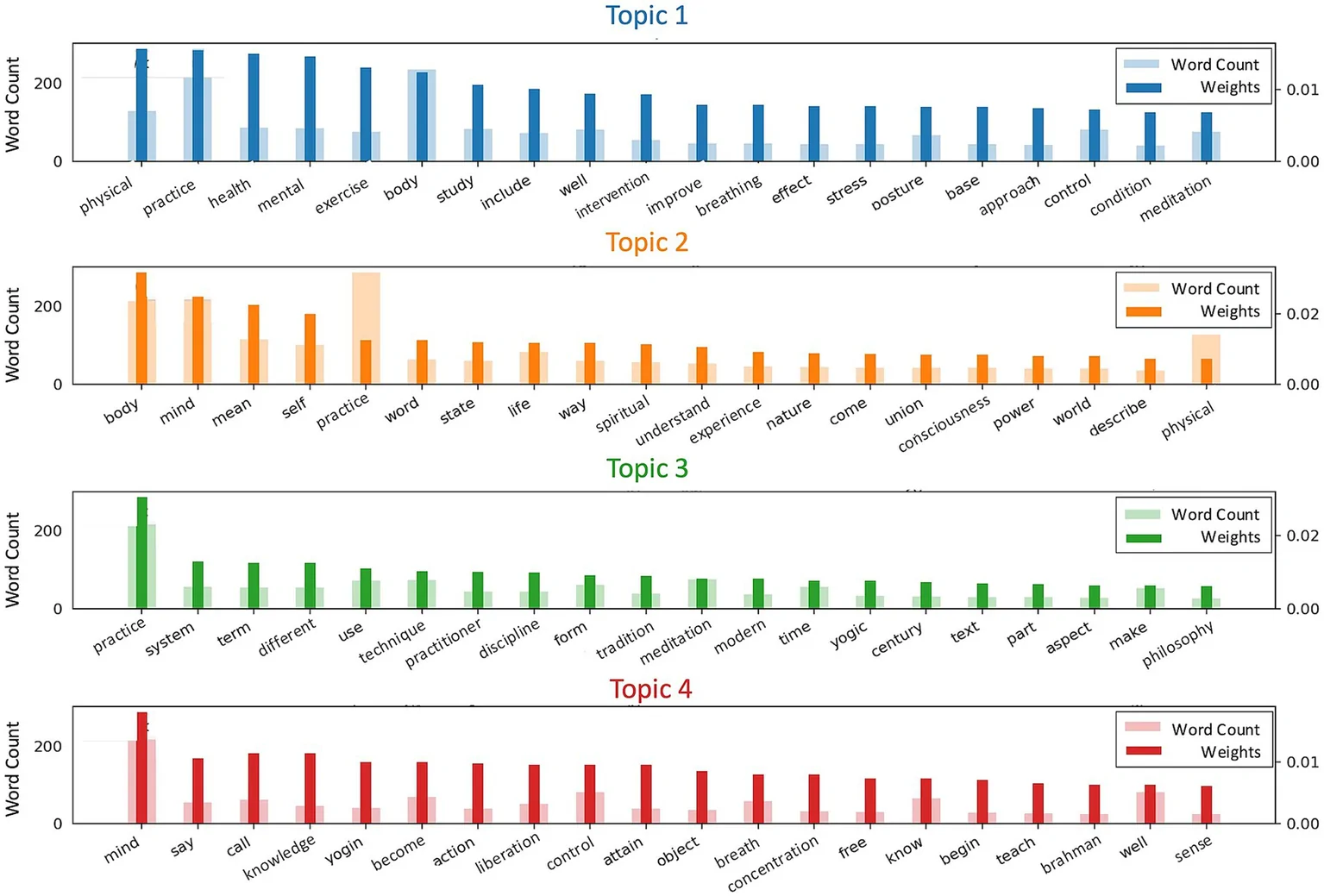
The frequency and importance of words in each topic. The word count and word importance of the top 20 keywords are shown for each of the four topics. Word count is how often the word occurs in the whole corpus. Word importance is indicated by the weight of the word loading on a topic, namely, the contribution/importance of the word to the topic. Only three words, body (topic 1), and practice and physical (topic 2) showed frequencies above their weight, suggesting they may be less specific to those topics. We note that physical, meditation, and body appeared in more than one topic. Topics are color-coded as in Figure 2B.

#### 2.3.4 Word cloud generation

The top probable words in each topic are displayed in Table 2. The top 20 most probable words in each topic were visualized using word clouds. Visualization was conducted in Python using the WordCloud library. Words were already tokenized and normalized to consolidate lexical variants. The resulting word cloud displayed the top 20 probable words in each topic, with the size of the keywords proportional to their importance weights (contributions) to the topic (Figure 4).

**Table 2:** Top 20 probable terms of topics in the unsupervised (LDAMallet) and semi-supervised (GuidedLDA) models.

Model used	Topic 1	Topic 2	Topic 3	Topic 4
LDAMallet λ = 1	Physical Health Mental Practice Study Exercise Include Posture Intervention Meditation Breathing Improve Effect Stress Base Condition Body Benefit Reduce Control	Body Mind Practice Life State Spiritual Understand Experience Nature Union Consciousness Individual Science Human Physical Path Soul Describe Achieve Divine	Practice Term Technique System Discipline Time Word Tradition Practitioner Modern Century Text Yogic Refer Meaning Part Context Liberation Practice Form	Mind Call Knowledge Yogin Action Attain Control Object Free Liberation Breath Sound Teach Begin Sense Brahman Sense Practice Result Good
GuidedLDA s = 0.468445 (best value)	Physical Practice Health Mental Exercise Body Study Include Well Intervention Improve Breathing Effect Stress Posture Base Approach Control Condition Meditation	Body Mind Mean Self Practice Word State Life Way Spiritual Understand Experience Nature Come Union Consciousness Power World Describe Physical	Practice System Term Different Use Technique Practitioner Discipline Form Tradition Meditation Modern Time Yogic Century Text Part Aspect Make Philosophy	Mind Say Call Knowledge Yogin Become Action Liberation Control Attain Object Breath Concentration Free Know Begin Teach Brahman Well Sense

**Figure 4:**
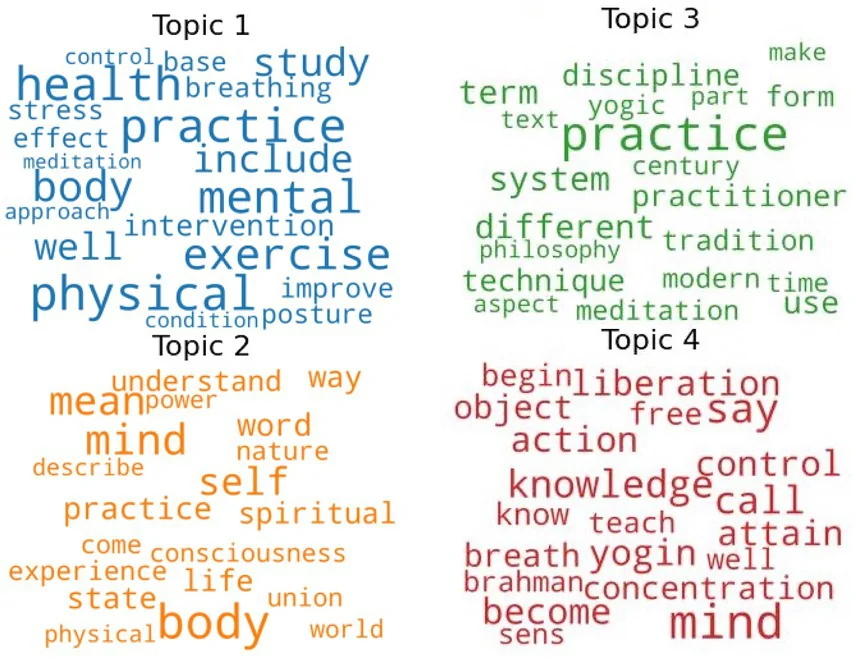
Word cloud of top 20 ranked terms in each topic. Word cloud plot of the top 20 probable words in each of the four topics, with the size of the keywords proportional to their importance weights (contributions) to the topic.

### 2.4 Post-statistical analysis

We used a series of statistical analyses that test whether the topics are congruent with the four source types used.

#### 2.4.1 Chi-squared test of independence

A chi-squared baseline analysis was used to test the null hypothesis that topic assignment was not significantly associated with the source type of the yoga definition. To do this, statistical analyses were performed in R using the stats library. A chi-squared test of independence was used to examine the relationship between [topic] and [source type]. After this test, we further plotted the standardized residuals, which revealed which topic-source type pairs were driving the association (Figures 5A,B; Table 3). Standardized residuals indicated that each topic was strongly associated with a distinct source type, suggesting that the model successfully captured meaningful thematic structure.

**Figure 5:**
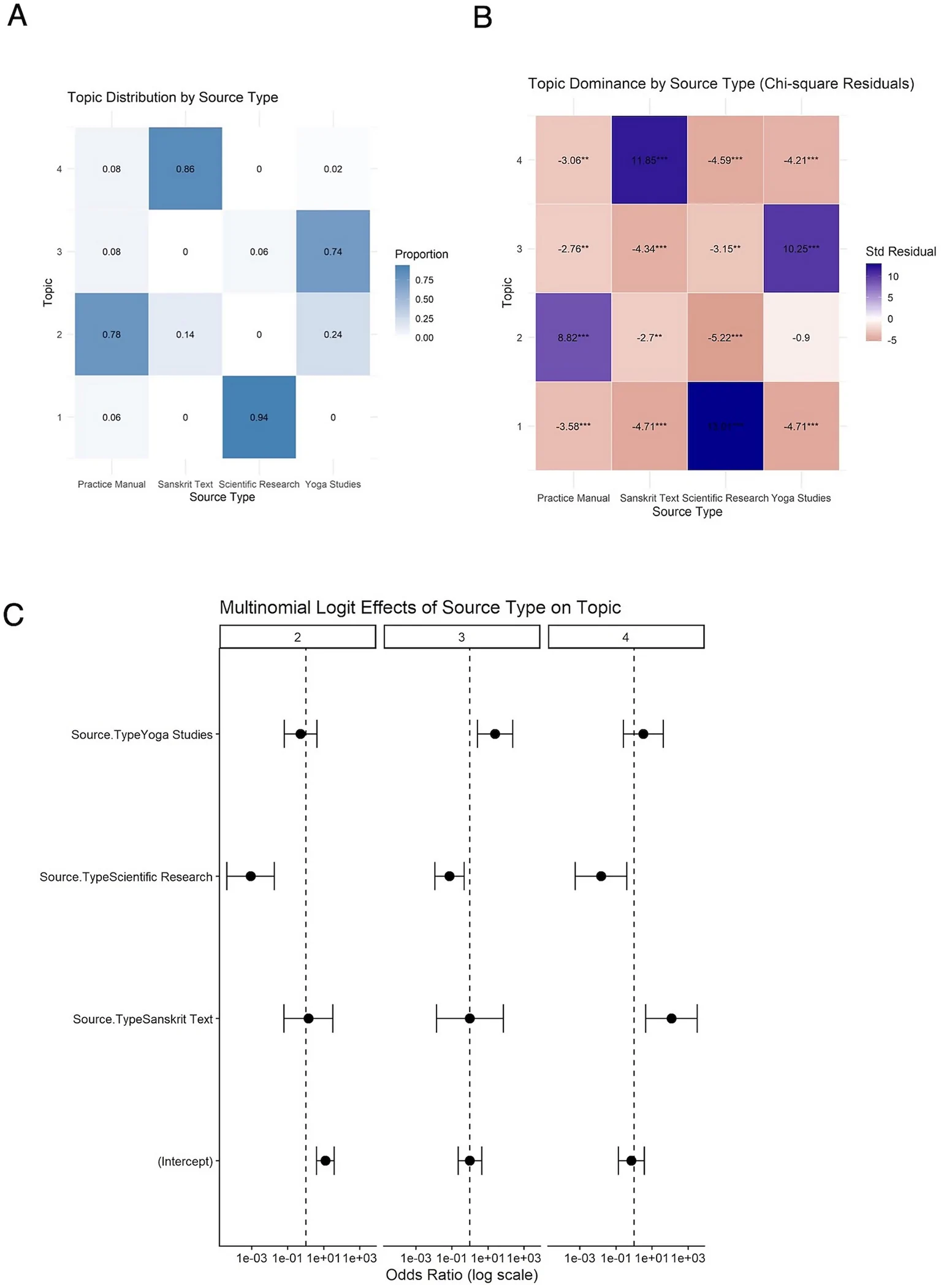
Statistical analysis indicates that topic dominance is related to source type in the baseline LDAMallet model and the SeededLDA/GuidedLDA model. Across the two models, a large proportion of the yoga definitions from scientific research discuss terms found in topic 1; practice manuals are linked to topic 2; academic yoga studies is connected to topic 3; and Sanskrit texts focus on topic 4. **(A)** shows topic distribution by source type in proportions for the semi-supervised model, while **(B)** shows chi-squared residuals (chi-squared = 377.87, df = 9, *p*-value < 2.2e-16), rejecting the null hypothesis and thus indicating that topic assignment is significantly associated with the corpus each yoga text belongs to. **(C)** Regression estimates in Odds Ratio Using Predicted Topic Labels from GuidedLDA Model supports the association of topics with source types. To clearly visualize the regression estimates of odds ratios and their significance, we plotted the odds ratio estimates of the coefficients using the predicted topic labels from the semi-supervised model. Significant effects were those in which the 95% confidence interval did not cover the odds ratio = 1 line.

**Table 3:** Semi-supervised model topic distribution by source type.

Topic	“Practice Manual”	“Sanskrit Text”	“Scientific Research”	“Yoga Studies”
Topic 1	3	0	47	0
Topic 2	39	7	0	12
Topic 3	4	0	3	37
Topic 4	4	43	0	1

#### 2.4.2 Penalized multinomial logistic regression

To further examine the probability of topic assignment based on source type and the change in probability of topic assignment as the source type changes, we used multinomial logistic regression (Figure 5C). The complication of this analysis was that the predictor source type largely predicts the topic assignment, leading to extremely large coefficients and standard errors caused by infinite maximum likelihood estimation. We thus used a specific function (brmultinom()) in R (using the brglm2 package) to fit the multinomial logistic regression using bias reduction methods and maximum penalized likelihood estimation using the power of Jeffreys prior as a penalty. We derived the estimates in both log odds ratio and odds ratio scales using the predicted topic labels from the baseline LDAMallet model (Supplementary Table 3A) as well as the semi-supervised SeededLDA/GuidedLDA model (Supplementary Table 3B).

#### 2.4.3 Average adjusted predictions and average marginal effects

We also derived the average predicted probabilities from the penalized multinomial logistic model to better understand the model using the “marginaleffects” library in R. We derived the average predicted probability of being assigned to each topic at each source type of yoga definition (Supplementary Tables 4A,B). We further derived the average marginal effects of source type on the predicted probability of being assigned to each topic rather than to all other topics (Supplementary Tables 5A,B).

#### 2.4.4 Independence of irrelevant alternatives (IIA)

The odds ratio of outcome m versus n is determined without reference to the other outcomes that might be available. This is the IIA assumption of multinomial logistic regression. We tested this assumption informally by dropping each outcome category, refitting the model, and checking whether coefficients for the remaining outcome categories change (Supplementary Table 6).

## 3 Results

### 3.1 Attributes of the source types analyzed

To analyze the corpora discussed above, we extracted 200-word text blocks that discuss what yoga is, selecting 50 from each of the four source types/corpora (Supplementary Table 1A). In some cases, the definition is not necessarily one written by the author, but rather their discussion of yoga definitions in other sources. We then checked the attributes of each of the four source types/corpora (summarized in Supplementary Table 1B). While the Sanskrit texts are largely from India, the other three corpora originate from a broader spectrum of countries. In addition, the source types were distributed across time, with Sanskrit texts representing an ancient corpus that spans through to the early modern era, and scientific and biomedical articles being the most contemporary sources.

### 3.2 Topic modeling analysis of yoga documents highlights four well-distinguished topics

#### 3.2.1 Initial analysis

Our collected data (Supplementary Table 1A) were prepared for NLP as described in the Methods. Removing extended stop words left a range of word counts between 60 and 160 terms in each yoga definition (Supplementary Figure 1A). The initial unsupervised model was run and visualized using an interactive pyLDAvis plot with different numbers of topics. Our initial analysis revealed that a four-topic model yielded coherent, well-separated topics (Supplementary Figure 2A). The bubbles were also evenly sized; the larger the bubble, the higher the percentage of documents in the corpus is about that topic. The even-sized bubbles indicate that the four topics are capturing roughly even-sized batches of the original dataset. Analysis of our yoga documents using t-SNE revealed well-separated clusters (Supplementary Figure 2B). Examining the terms within each cluster revealed four preliminary themes based on our domain knowledge (Supplementary Figure 2C).

#### 3.2.2 Semi-supervised topic modeling

Since the unsupervised LDA model can be prone to creating topics that are not meaningful (Chang et al., 2009), we used a semi-supervised topic model (SeededLDA/GuidedLDA), which allowed us to apply domain knowledge to seed the models (Andrzejewski et al., 2009; Andrzejewski and Zhu, 2009; Gallagher et al., 2017; Jagarlamudi et al., 2012; Yang et al., 2015; Zhai et al., 2011).

We chose anchor terms based on the most distinctive/unique terms in each topic as visualized in pyLDAvis, selecting the top-ranked terms with λ = 0, a setting that reveals topic-distinct words. The resulting anchor terms (Table 1) were used to guide the semi-supervised analysis. The semi-supervised model with four distinct topics (Figure 2A) was independently validated using t-SNE (Figure 2B). While the four topics are distributed differently from the unsupervised model, they remain well-separated and contain very similar terms to those from the unsupervised model. This may indicate that the unlabeled data contain a strong, clear topic structure.

### 3.3 Analysis of keywords associated with the four topics and underlying themes

Next, we examined the top keywords for each topic to identify underlying themes. Examining the top probable keywords reveals that the unsupervised and semi-supervised models overlap (Table 2). Thus, the four topics remained stable across analyses and well-distributed, though their exact distribution on the pyLDAvis shifts (Figure 2; Supplementary Figure 2A). We focus on output from the semi-supervised model from here onward.

We analyzed the top-20 keywords for each topic uncovered by the semi-supervised model, examining their frequency of occurrence in the corpus and their importance (weight) within each of the four topics (Figure 3). This approach allowed us to contrast word count with word importance for each keyword in each topic.

Word clouds for each topic indicate visually that the four topics have distinct vocabularies, with only a very few words that appear in more than one topic (Figure 4). Keywords in topic 1, that also have robust weight relative to frequency (Figure 3) include physical, practice, health, mental, exercise, breathing, stress, and control. The focus in this topic seems to be concrete and interventionist techniques related to yoga, and so we define this topic as an interventionist/outcome cluster. The highest-ranked keywords in topic 2, body, mind, and mean were not by themselves informative, but the topic contains many terms that suggest a more experientially inflected understanding of yoga practice, such as self, experience, spiritual, union, way, life, nature, power, and consciousness. The focus here is less on practical effects and meanings of yoga and more on an experiential understanding of yoga. We term this the experiential cluster. Terms such as philosophy, century, modern, system, practitioner, tradition, and technique suggest that topic 3 leans towards historical and critical analysis of the transmission of specific lineages of yoga tradition and practice. We call this the genealogical cluster. Finally, topic 4 contains terms that suggest soteriological and spiritual ideas, such as mind, brahman, knowledge, yogin, and liberation, as well as terms that suggest specific methods for plausibly accessing these spiritual states, including control, attain, object, breath, and concentration. While overlapping with topic 2 in its focus on spiritual states, topic 4 also seems to include terms related to particular mental practices and disciplines through which spiritual states can be acquired. We define topic 4 as the soteriological cluster.

### 3.4 The four topics indicate the underlying grammar of each corpus

Having examined keywords for each topic, we analyzed which documents were contributing to each topic. We first examined whether the four corpora might underlie the four topics, using a chi-squared analysis to test the null hypothesis that topic assignment is not significantly associated with source type/corpus. Our data rejected this null hypothesis, indicating that topic assignment is significantly associated with the source type of yoga definition (chi-squared = 383.72, df = 9, *p*-value < 2.2e-16 for the unsupervised model and chi-squared = 377.87, df = 9, *p*-value < 2.2e-16 for the semi-supervised model).

While the chi-squared test assumes cell counts ≥ 5, and we had some cell counts that were 0, the signal is very strong, indicating the robustness of this conclusion. After this test, we plotted the standardized residuals, which again showed that topic-source type pairs were driving the association, with distinct source types dominating certain topics (Figure 5A shows topic distribution by source type by proportion for the semi-supervised model). Standardized residuals indicated that each topic was strongly associated with a distinct source type, suggesting that the model successfully captured a meaningful thematic structure in the data (Figure 5B; chi-squared = 377.87, df = 9, *p*-value < 2.2e-16 for the semi-supervised model).

To further examine the probability of topic assignment based on source type and the change in probability of topic assignment as source type changes, we used multinomial logistic regression. Since the predictor source type largely predicts the topic assignment, we saw extremely large coefficients and standard errors caused by infinite maximum likelihood estimation. We thus fit a multinomial logistic regression model using bias-reduction methods and maximum penalized likelihood estimation with a penalty (power of Jeffreys prior). The resulting regression estimates validated a relationship between pairs of specific topics and source types for both the unsupervised and semi-supervised models (Supplementary Table 3). Interpretation of the coefficients from these analyses is provided in the Supplementary methods. To more clearly visualize these effects and their significance, we further plotted the odds ratio estimates of coefficients (Figure 5C) using the predicted topic labels from the semi-supervised model.

Next, we derived the average predicted probability of assignment to each topic for each yoga-definition source type using a penalized multinomial logistic model (Supplementary Table 4). The average predicted probabilities strongly support topic 1 (yoga interventions/outcomes) being associated with the scientific research corpus (0.91346 (95% CI: [0.83553, 0.9914])). Meanwhile, topic 2 (phenomenological) is highly associated with practice manuals (0.75962 (95% CI: [0.64117 0.8781])). Topic 3 (historical/analytic) is associated with yoga studies (0.72115 (95% CI: [0.59686 0.8455])) and topic 4 (soteriological) relates to the Sanskrit text corpus (0.83654 (95% CI: [0.73404 0.9390])). Analysis of the change in predicted probability of topic assignment as source type changes, marginal effects analysis, further confirmed these assignments (Supplementary Table 5).

Finally, we dropped each outcome category in turn, refit the model, and asked whether the analysis outcome changed (the IIA assumption of multinomial logistic regression; Supplementary Table 6). When dropping topic 2, the odds ratios of topic 3 vs. topic 1 and topic 4 vs. topic 1 were unchanged. The same occurred when dropping topic 3 or topic 4. When topic 1 was dropped, the results shifted. However, since the baseline outcome category (topic 1) remains the same across contrasting models when dropping alternative outcomes (topics 2, 3, or 4), overall, the pairwise relationship between topic assignments and distinct corpora holds (Supplementary Table 6).

Together, these post-statistical analyses indicate that there is a high probability of scientific research into yoga contributing to topic 1 (yoga interventions/outcomes), yoga practice manuals being linked to topic 2 (phenomenological terms), academic yoga studies being connected with topic 3 (historical/analytic), and Sanskrit texts being associated with topic 4 (soteriological). These associations are further supported by the number of yoga documents from each corpus that are found in each topic (Table 3). Overall, mapping topics onto specific corpuses shows that although texts within each corpus may differ as lineages and traditions evolve, each corpus has an internally robust grammar.

### 3.5 Notable texts that associate with the four topics

We then examined notable texts that are associated with the dominant topic for their source types. To do this, we examined the top 10 ranked yoga texts associated with each of the four topics based on their percentage contributions to that topic (Supplementary Table 7). We also examined texts that were “atypical” in that they were associated with a topic that is distinct from the rest of this corpus (Supplementary Table 8).

#### 3.5.1 The interventionist/outcome cluster (topic 1)

The scientific texts formed a tight group that falls into the intervention/outcome topic (Table 3), as might be expected. These texts emphasize a mixture of psychological and postural terms to articulate the beneficial effects that yoga might have for various health conditions. However, three research papers aligned more closely with the historical/analytic group (aligning with the academic yogic studies corpus; Supplementary Table 8). One of these papers (Malone et al., 2025) was among the few qualitative analyses logged in PubMed, so it entered the study as part of the biomedical collection. The paper uses a Black feminist approach to ground its exploration of yoga practices. Clustering with the academic yoga studies corpus is not completely a surprise, given that the authors take a critical-historical approach to the study of yoga and explicitly state that “It would be a negligent Black feminist practice not to re-member yoga’s indigeneity to South Asia, given how colonialism, appropriation, consumerism, and exoticization have impacted yoga’s millennia of evolution” (Malone et al., 2025, p. 2).

Additionally, three yoga practice manuals were grouped with the cluster of scientific texts. One of these is Indra Devi’s Yoga for Americans, famous for tying the benefits of yoga practice to women’s health. In her definition of yoga, she explicitly presents yoga as a unique practice of health education: “The science of yoga has a separate division devoted to the most thorough care of the human body and all of its functions from breathing to elimination. These methods are entirely different from other methods of health education because yoga aims first of all to remove the very causes of ill health which are brought about by insufficient oxygenation, poor nutrition, inadequate exercise, and poor elimination of the waste products that poison the system. Secondly, through rhythmic breathing and concentration, as well as by influencing our glandular activity, yoga can help us to increase our mental capacities, sharpen our senses and widen our intellectual horizon and finally through meditation it enables man to come closer to the realization of his own spiritual nature.” (Devi, 1959, p. xi) Even when Devi mentions the idea of “spiritual nature,” it is more of a cursory reference rather than the part of a more integrated spiritual grammar that we find in the clusters related to yoga manuals and the classical canon of yoga.

#### 3.5.2 The experientially oriented cluster (topic 2)

Even though it is relatively less concentrated in a specific source type than topic 1 (Figure 5), this topic paired well with the corpus of practice manuals, which formed the bulk of the texts in this category. This is not altogether surprising because practice manuals of yoga tend to frame yoga as a spiritual and experiential practice even as they ground these in specific anatomical postures and exercises. A representative example of a practice manual for this topic is Doug Keller’s manual on hatha yoga that still defines yoga while saying, “Yoga in all its forms is itself the endeavor of the creative power of the universe Spirit to see and know Her own Beauty through us, Her creation. Through yoga we awaken from our own individual and limited perceptions to the Beauty and Truth of the whole, of ourselves as Spirit and the manifestations of that one Spirit in countless forms. Hatha Yoga is one such practice within the Pantheon of Yoga; its asanas or poses bring us to experience and express the integrity of the body as a whole according to its own energetic form, moving us from our immersion in our physical life toward experiencing the higher creative intelligence of the universe at work within us” (Keller, 2004, p. 15). This is resonant with yoga studies scholar Suzanne Newcombe’s argument that modern yoga, as exemplified in yoga manuals and yoga studios, is not just represented as a postural practice, even if it might have become so in practice (Singleton, 2010), but is rather formed by hybridizing different frameworks, spiritual and secular, to frame the practice of yoga (Newcombe, 2019).

Given that this topic is more loosely paired with the specific corpus of practice manuals than was the case for topic 1, there is a larger number of texts from other sources that are paired with this topic, most notably texts from classical treatises on yoga and texts from the corpus of yoga studies. An example of the former would be the *Kaṭha Upaniṣad,* which provides a strong metaphysical description of yoga. An example of a text from the corpus of yoga studies that strongly pairs with the topic are lectures on yoga given by the prominent social reformer Keshub Chandra Sen (Sen, 1904). The inclusion of both these texts suggests that this topic generally pairs well with spiritually inflected definitions of yoga, regardless of what the rest of the text might include. This explains some of the overlap in this topic between the more concrete and practical yoga manuals and some texts from other corpora that might lack a practical component entirely.

#### 3.5.3 The genealogical cluster (topic 3)

The third topic pairs well with the more academically oriented studies of yoga (Table 3), where the focus is a more historically and genealogically inclined specification of yoga traditions and practices. Some famous exceptions in this corpus are the work of the prominent anthropologist, Joseph Alter, whose description of yoga as a form of ‘nature cure’ paired more strongly with topic 2 than topic 3 (Alter, 2018). However, this becomes more explicable when one acknowledges that Alter was reproducing a description of yoga that his interlocutors provided as opposed to providing one of his own. This brings up a larger point in relation to topic 3. While most of the yoga studies scholars included in this corpus provided their own framework to describe yoga, there were some who were reproducing or referencing the descriptions of yoga they found in the texts or practices of yoga that they were analyzing. This conflation might have caused some of the yoga studies texts to pair with different topics, as is the case with Alter above. But the association between this topic (topic 3) and the yoga studies corpus is strong enough to suggest a corpus-specific concentration on this topic.

#### 3.5.4 The soteriological cluster (topic 4)

This cluster mixes terms for spiritual states with a more contemplative grammar around methods of meditation. While descriptions of spiritual states are shared between topic 2 and topic 4, a focus on the set of terms related to contemplative method distinguishes topic 4 from topic 2. There is a very strong association between topic 4 and the corpus of classical texts. This association is important as it possibly suggests that a contemplative grammar might have had a diffuse influence on the broader landscape of yoga despite its multifarious lineages. Notably, one yoga studies and four practice manuals paired with this topic; the latter included the *Yoga Makaranda* by Tirumalai Krishnamacarya, a yoga manual by Bikram Choudhary, AC Bhaktivedanta Swami Prabhupāda’s work, and guidance on yoga from Sri Ramana Maharshi (Bhaktivedanta Swami Prabhupāda, 1972; Choudhury and Reynolds, 2000; Krishnamacharya, 2006; Venkataramiah, 2006). The pairing of these texts with the classical corpus indicates that certain sub-traditions of yoga practice might blend contemplative and spiritual aims more strongly than some others. However, the topic models only capture this information in a weak manner.

## 4 Discussion

By analyzing data containing discussion of what yoga is from four corpora of yoga texts, we have identified four topics that seem to represent distinct grammars related to yoga. While this might be intuitively expected, our manuscript identifies these semantic fields using a bottom-up, inductive, and computational approach. We use grammar in a Wittgensteinian sense to suggest that a term only gains meaning in the network or neighborhood of other words in which a term seems to be recurrently present (Wittgenstein, 1958). In this sense, the four topics reflect four internally networked grammars of yoga rather than isolated clusters of terms. Topic 1 constitutes a grammar of yoga that is more interventionist and outcome-oriented. Topic 2 constitutes a grammar of yoga that is grounded in spiritual states. Topic 3 suggests a classificatory and scholarly grammar that is more genealogically oriented. Finally, Topic 4 suggests a grammar that is a hybrid of contemplative practices and spiritual states. These grammars have strong associations with the four topics that were extracted from the database, thereby suggesting that a robust topic structure is present in our dataset of yoga definitions. This has several implications for the study of yoga, discussed below.

### 4.1 Four grammars of yoga rather than a unitary definition

The approach used here might have identified topics that cross corpora: for example, discussion of the etymology of yoga from the Sanskrit verbal root √*yuj* is not restricted to the Sanskrit corpus, but appears in 20 documents across the corpora. Some frequent terms, including practice, mind, physical, and, meditation appeared in multiple topics, suggesting they may reflect attributes of yoga that apply across topics. We found that certain terms, such as body (in topic 1), practice and physical (in topic 2), and meditation (topic 3), have a high frequency compared to weight (Figure 3), indicating low importance to the specific theme of the topic. However, the model, even when unsupervised, did not nucleate topics around these terms; instead, it separated thematic grammars that turned out to be distinct for each corpus. This outcome indicates that there are no core unitary definitional structures but rather four grammars around which our database of yoga definitions organizes itself. Furthermore, each of these grammars have their own differentiated way of emphasizing the psychological aspects of yoga. Topic 1 uses a well-specified health-oriented vocabulary of stress, anxiety, mind, meditation, and control within which the psychological aspects of yoga are grounded. Topic 2, on the other hand, does not specify the psychological aspects of yoga as specific psychological states, but leveraging its grounding in a more experiential, even phenomenological, understanding of inner states of being to draw out the psychological implications of yoga. An example of a description of such inner states ‘harmony’ in the Iyengar school of yoga or ‘alignment’ in the Kaivalyadhama school of yoga. Topic 4 also bypasses a discussion of specific psychological states in favor of a set of terms that pertain either to contemplative, even cognitive, practice of yoga or to the ultimate spiritual reality that can be accessed through such practices of yoga. For the classical texts associated with this topic, a discussion of intermediate and accompanying psychological states seems less important than specifying the contemplative mechanisms to attain liberation.

One further implication of our analysis is that, if we were to, for example, expand the sub-corpora, by including 50 definitions from different modern postural schools of yoga, we would possibly find that each of the schools employs its own sub-grammar of yoga. This idea is hinted at in the practice manuals of Desikachar and Cravens (1998) and Kraftsow (1999), in the Viniyoga lineage of modern yoga, which are independently associated with a topic otherwise dominated by academic publications in the field of yoga studies. Currently, our database does not meaningfully capture the internal heterogeneity within any of the four topics. Future work would be needed to test if sub-corpora do, in turn, possess their own differentiated grammars.

### 4.2 The canalizing effects of institutional contexts

The presence of these distinct grammars arises, perhaps, from the language that is most acceptable to the institutional location most closely associated with that corpus. In other words, the grammatical diversity of yoga might also map well onto the institutional diversity within which the practice of yoga is conducted. The institutions of the modern yoga studio, the scientific enterprise, and the university setting, thus, tend to favor grammars that analyze or pivot the meaning of yoga to make it legible to their own particular audiences. Thus, practical manuals favor subjective experience of the practitioner, the scientific enterprise focuses on diseases such as stress and interventions, while academic institutional actors favor historical analysis of specific sub-traditions of yoga practice. While some modern postural schools of yoga might focus on the contemplative and soteriological potential of yoga that is central to Sanskrit texts, for the most part the global expansion of yoga has, as many scholars have noted, led to the re-rendering of these ideas and a focus on the phenomenological experiences of yoga even if these experiences are framed in a generic spiritual register. It is important to acknowledge this inadvertent centrality of institutional setting for the knowledge production of yoga for any meaningful dialogue between yoga scholars, practitioners, researchers, and teachers to take place.

### 4.3 The implications for scientific analysis and interventions

The analysis also has significant implications for the scientific measurement and assessment of the outcomes of yoga, in psychology and more broadly. Scientific articles might acknowledge the contemplative or spiritual aspects of yoga in a cursory way by stating that, “Yoga is a multifaceted mind-body discipline originating in India, encompassing physical postures (*āsanas*), breathing techniques (*prāṇāyāma*), meditation and mindfulness (*dhyāna*), and relaxation practices” (Giridharan et al., 2025). These acknowledgments, however, tend to be disconnected from the more specific operationalizations of yoga that they employ as interventions in their study. Our analysis suggests that, given the stickiness of the respective grammar to each of the four corpuses of yoga, even partially integrating the spiritual and contemplative grammar of yoga is considerably more challenging. Instead of trying to assimilate contemplative, pedagogical, historical, or community-based vocabularies into a single scientific framework, a more productive dialogue may involve better specifying the practice of yoga being studied, acknowledging the historical and institutional contexts of different accounts of yoga, documenting partial correspondences, and preserving meaningful differences where equivalence would be reductive.

Most scientific studies of yoga also rely on a limited and uniform set of protocols even as they often present the findings of their studies as being about yoga more generally (for example, Bowden et al., 2012; Brown and Gerbarg, 2005; Büssing et al., 2012; Fiori et al., 2014; Gard et al., 2013; Shapiro and Cline, 2004). Instead of employing a manual from a particular lineage or tradition of yoga which would cause copyright and standardization issues, scientists often create a standardized experimental protocol in collaboration with advanced yoga teachers and practitioners, rather than employing a manual from a particular lineage or tradition of yoga (Kumar et al., 2021; Nagendra and Gangadhar, 2026; Ramirez-Duran et al., 2025). While such an approach avoids the conundrums of cherry-picking a tradition of yoga to test and creates standardized protocols of yoga that can be cross-tested, this can have overgeneralizing effects given the conceptual and practical variation between different schools of yoga. Integrating the concepts from the more contemplative and spiritual grammars of yoga, such as Aurobindo (1992) idea of integration, Kuvalayananda (1963) idea of alignment, and Iyengar and Rivers-Moore (1988) idea of harmony into the standardized protocol is even more difficult than just including the *asanas* from different sub-traditions of yoga. While some recent scientific scholarship has started taking some steps in this direction by drawing on concepts from more positive psychological approaches to understand the mediating effects of yoga, such as psychological flexibility (Kashdan and Rottenberg, 2010), flow states (Butzer et al., 2016), or eudaimonic well-being (Ivtzan and Papantoniou, 2014; Sullivan et al., 2018), a more philosophically rich and indigenously engaged yoga therapy is still a highly aspirational enterprise. Finally, even if yoga protocols are standardized, this might not match the phenomenological experiences of people who engage in yoga as recipients of the health interventions. This is necessary to not only create a comprehensive account of the mental and contemplative states that often accompany the practice of yoga but also to get a more fine-grained understanding of the mechanisms behind the positive outcomes of yoga on health.

### 4.4 Limitations and future work

Our work has several limitations. First, our paper recognizes that definitions of yoga are often minor proxies for the intentions of the author and the totality of the text from which the definitions of yoga were extracted. To achieve a more fine-grained analysis of any particular grammar or sub-grammar of yoga, future work could experiment with the number of texts to include in the pipeline and the number of tokens to include from each text to achieve a more comprehensive representation of each text in the corpus. Second, the comparative scope of our paper is limited by the number of texts we could include from each corpus. While 50 classical sources of yoga is a substantive number to include in a database of yoga texts, 50 scientific studies or 50 yoga manuals are not. While our conclusion pertains specifically to the limited sample of texts in our dataset, our hope is that future work will expand the corpora to enable larger-scale studies that go beyond the necessary selections made for this study. For example, future work could compare the biomedical literature across different decades or yoga manuals from distinct countries. This will significantly expand our understanding of the heterogenous sub-grammars of each corpus of yoga texts. Third, our analysis of the Sanskrit corpus is considerably restricted by the small number of robust translations that are available, and future work should include translations of sources in additional Indic languages. Additionally, the act of translating Sanskrit terms into legible English analogs is a fraught process and might not fully reflect the original intent or semantic range of the original Sanskrit term. However, this is a necessary cost of any analysis that compares texts across different languages. Finally, while topic models provide a good bird’s-eye view of the different themes that structure the corpus of yoga, it lacks contextual granularity. Finally, it is also possible that other text types or sub-corpuses can be defined, and we hope our study points the way to analysis.

Our study computationally maps the semantic dimensions of four distinct grammars of yoga but future, more qualitatively oriented textual studies that include close readings of specific texts will provide rich information on how this grammar maps onto specific institutional, historical, and cultural settings. We alluded to some of these possibilities while providing brief discussions of individual yoga texts above, but we hope that our paper at least provides a starting point for much more in-depth future work on the overlaps and differences between the four grammars of yoga.

## 5 Conclusion

In this exploratory study, we analyzed 200 English-language passages drawn from four purposively constructed source domains: translated premodern Sanskrit sources, modern yoga practice manuals, academic scholarship, and biomedical research articles. The models identified four recurring lexical orientations concerning health and intervention, experiential and spiritual practice, historical and genealogical analysis, and contemplative or soteriological aims. Our analysis shows that there are four distinct topics that organize the semantic field of yoga with each topic pairing strongly with one of the four corpora of yoga definitions. In providing this bird’s-eye view, our paper provides empirical validation to show that our sample literature on yoga is fragmented into four sufficiently differentiated textual universes, each with its own distinctive organizing grammar that determines the boundary between those concepts and terms that make sense to include in that grammar and those that do not. This fragmentation of grammars across different yoga corpora reveals the difficulty of creating even a partially unified field of yoga studies that integrates concepts and ideas from different perspectives (philosophical, pedagogical, scholarly, and scientific) used to study the psychophysical aspects of yoga. Scholar-practitioners may well anticipate that these literatures diverge. What our paper adds is a more comprehensive account of what networks of concepts might divide them, with the distinguishing terms named and weighted (Table 2, Figure 3), along with the distance between the separate topics (Figure 2), with the change in topic probability across source types estimated and bounded (Supplementary Tables 4, 5). The strong association we found between the source type and the respective grammar in our sample, however, should not be seen as a sign of the impossibility of dialogue across different stakeholders in the field of yoga studies, but as an articulation of the space of possibility for future work that aims to create a dialogically plural and mutually enriching field of yoga studies.

## Data Availability

The original contributions presented in the study are included in the article/Supplementary material, further inquiries can be directed to the corresponding author/s.

## Footnotes

1
http://hyp.soas.ac.uk/

## References

[ref1] Alter J. S. (2018). “Yoga, nature cure and “perfect” health: the purity of the fluid body in an impure world,” in Yoga in Transformation: Historical and Contemporary Perspectives, eds. Baier K. Maas P. A. Preisendanz K. (Göttingen: V&R unipress), 419–439.

[ref2] Andrzejewski D. Zhu X. (2009) Latent Dirichlet allocation with topic-in-set knowledge Proceedings of the NAACL HLT 2009 Workshop on Semi-Supervised Learning for Natural Language Processing, SemiSupLearn’09 USA: Association for Computational Linguistics 43–48

[ref3] Andrzejewski D. Zhu X. Craven M. (2009) “Incorporating domain knowledge into topic Modeling via Dirichlet Forest priors,” in Proceedings of the 26th Annual International Conference on Machine Learning, ICML’09 New York: Association for Computing Machinery 25–32 10.1145/1553374.1553378 PMC2943854 20871805

[ref4] Aurobindo Ś. (1992). The Synthesis of Yoga. Twin Lakes, USA: Lotus Press.

[ref5] Bhaktivedanta Swami Prabhupāda A. C. (1972). The Perfection of Yoga. New York: ISKCON Press.

[ref6] Blei D. M. Ng A. Y. Jordan M. I. (2003). Latent dirichlet allocation. J. Mach. Learn. Res. 3, 993–1022.

[ref7] Bowden D. Gaudry C. An S. C. Gruzelier J. (2012). A comparative randomised controlled trial of the effects of brain wave vibration training, Iyengar yoga, and mindfulness on mood, well-being, and salivary cortisol. Evid. Based Complement. Alternat. Med. 2012:234713. doi: 10.1155/2012/234713, 22216054 PMC3246835

[ref8] Brown R. P. Gerbarg P. L. (2005). Sudarshan kriya yogic breathing in the treatment of stress, anxiety, and depression: part I—neurophysiologic model. J. Altern. Complement. Med. 11, 189–201. doi: 10.1089/acm.2005.11.189, 15750381

[ref9] Büssing A. Hedtstück A. Khalsa S. B. S. Ostermann T. Heusser P. (2012). Development of specific aspects of spirituality during a 6-month intensive yoga practice. Evid. Based Complement. Alternat. Med. 2012:981523. doi: 10.1155/2012/981523, 22852023 PMC3407840

[ref10] Butzer B. Ahmed K. Khalsa S. B. S. (2016). Yoga enhances positive psychological states in young adult musicians. Appl. Psychophysiol. Biofeedback 41, 191–202. doi: 10.1007/s10484-015-9321-x, 26721471 PMC4860116

[ref11] Chang J. Boyd-Graber J.L. Gerrish S. Wang C. Blei D.M. (2009). Reading tea leaves: how humans interpret topic models Advances in Neural Information Processing Systems. Presented at the Advances in Neural Information Processing Systems 22: 23rd Annual Conference on Neural Information Processing Systems 2009, (Vancouver, BC: Neural Information Processing Systems) pp. 288–296

[ref12] Choudhury B. Reynolds B. J. (2000). Bikram’s Beginning Yoga Class: Revised and Updated. 2nd Edn Los Angeles: Tarcher / Penguin.

[ref13] De Michelis E. (2007). A preliminary survey of modern yoga studies. Asian Med. 3, 1–19. doi: 10.1163/157342107x207182

[ref14] Desikachar T. K. V. Cravens R. H. (1998). Health, Healing, and beyond: Yoga and the Living Tradition of Krishnamacharya. New York: Aperture.

[ref15] Devi I. (1959). Yoga for Americans: A Complete 6 Weeks’ Course for Home Practice. Englewood Cliffs: Prentice-Hall.

[ref16] Fiori F. David N. Aglioti S. M. (2014). Processing of proprioceptive and vestibular body signals and self-transcendence in ashtanga yoga practitioners. Front. Hum. Neurosci. 8:734. doi: 10.3389/fnhum.2014.00734, 25278866 PMC4166896

[ref17] Foxen A. P. Kuberry C. (2021). “What is yoga?” in Is This Yoga?: Concepts, Histories, and the Complexities of Modern Practice, (London: Taylor & Francis).

[ref18] Gallagher R. J. Reing K. Kale D. Ver Steeg G. (2017). Anchored correlation explanation: topic modeling with minimal domain knowledge. Trans. Assoc. Comput. Linguist. 5, 529–542. doi: 10.1162/tacl_a_00078

[ref19] Gard T. Brach N. Holzel B. Noggle J. Conboy L. Lazar S. (2013). Effects of a yoga-based intervention for young adults on quality of life and perceived stress: the potential mediating roles of mindfulness and self-compassion. J. Posit. Psychol. 7, 165–175. doi: 10.1080/17439760.2012.667144, 37339054

[ref20] Giridharan S. Shanbhag N. M. Bin Sumaida A. (2025). Effects of yoga interventions on health outcomes in women with gynecological cancers: a systematic review of randomised controlled trials. Cureus 17:e95017. doi: 10.7759/cureus.95017 PMC12630997 41281133

[ref21] Govindaraj R. Karmani S. Varambally S. Gangadhar B. N. (2016). Yoga and physical exercise - a review and comparison. Int. Rev. Psychiatry 28, 242–253. doi: 10.3109/09540261.2016.1160878, 27044898

[ref22] Ivtzan I. Papantoniou A. (2014). Yoga meets positive psychology: examining the integration of hedonic (gratitude) and eudaimonic (meaning) wellbeing in relation to the extent of yoga practice. J. Bodyw. Mov. Ther. 18, 183–189. doi: 10.1016/j.jbmt.2013.11.005, 24725784

[ref23] Iyengar B. K. S. Rivers-Moore D. (1988). The Tree of Yoga: Yoga Vṛkṣa, Shambhala Classics. Boston: Shambhala.

[ref24] Jagarlamudi J. Daumé H. Udupa R. (2012) Incorporating lexical priors into topic models Proceedings of the 13th Conference of the European Chapter of the Association for Computational Linguistics, EACL’12 USA: Association for Computational Linguistics 204–213

[ref25] Jain A. (2014). Selling Yoga: From Counterculture to Pop Culture. Oxford: Oxford University Press.

[ref26] Kashdan T. B. Rottenberg J. (2010). Psychological flexibility as a fundamental aspect of health. Clin. Psychol. Rev. 30, 865–878. doi: 10.1016/j.cpr.2010.03.001, 21151705 PMC2998793

[ref27] Keller D. (2004). Anusara Yoga: Hatha Yoga in the Anusara Style. 3rd Edn South Riding: DoYoga Productions.

[ref28] Kraftsow G. (1999). Yoga for Wellness: Healing with the Timeless Teachings of Viniyoga. New York: Penguin Books.

[ref29] Krishnamacharya T. (2006). Yoga Makaranda. Chennai: Krishnamacharya Healing & Yoga Foundation.

[ref30] Kumar V. Jagannathan A. Bhargav H. Varambally S. Gangadhar B. N. (2021). Generic yoga modules for clinical and research settings-need of the hour. Int. J. Yoga Ther. 31:25. doi: 10.17761/2021-D-18-00016, 34260713

[ref31] Kuvalayananda S. (1963). Yogic Therapy: Its Basic Principles and Methods. New Delhi: Central Health Education Bureau, Government of India.

[ref32] Mallinson J. Singleton M. (2017). Roots of Yoga. London: Penguin Books Limited.

[ref33] Malone N. Palomino K. A. Penniston T. L. Adane N. Kerney M. A. Hargons C. N. (2025). “It’s an honor to be in this body”: a black feminist grounded theory of embodiment through yoga among black womxn. Body Image 55:101982. doi: 10.1016/j.bodyim.2025.101982, 41101089

[ref34] Nagendra H. R. Gangadhar B. N. (2026). “Foreword,” in Good Yoga Practices: A Manual of Yogic Techniques, ed. Anand A. (Singapore: Springer).

[ref35] Newcombe S. (2018). “Spaces of yoga - towards a non-essentialist understanding of yoga,” in Yoga in Transformation: Historical and Contemporary Perspective, eds. Baier K. Maas P. A. Preisendanz K. (Göttingen: V&R University Press), 551–573.

[ref36] Newcombe S. (2019). Yoga in Britain: Stretching Spirituality and Educating Yogis. Sheffield: Equinox.

[ref37] Newcombe S. O’Brien-Kop K. (2021). Routledge Handbook of Yoga and Meditation Studies. London: Routledge.

[ref40] Ramirez-Duran D. Kern M. L. Stokes H. (2025). From practice to lifestyle: conceptualizations of yoga in regular ashtanga yoga practitioners using reflexive thematic analysis. Front. Psychol. 16:1582275. doi: 10.3389/fpsyg.2025.1582275, 40538480 PMC12177368

[ref42] Sarbacker S. R. (2021). Tracing the Path of Yoga: The History and Philosophy of Indian Mind-Body Discipline. Albany: Suny Press.

[ref43] Schmalzl L. Jeter P. Khalsa S. B. S. (2021). “The psychophysiology of yoga,” in Routledge Handbook of Yoga and Meditation Studies, ed. Newcombe S. (Abingdon: Routledge), 440–459.

[ref44] Sen K. C. (1904). Keshub Chunder Sen’s Lectures in India. London: Cassell and Company.

[ref45] Shapiro D. Cline K. (2004). Mood changes associated with Iyengar yoga practices: a pilot study. Int. J. Yoga Ther. 14, 35–44. doi: 10.17761/ijyt.14.1.47r6323xv20h786u

[ref46] Singleton M. (2010). Yoga Body: The Origins of Modern Posture Practice. Oxford: Oxford University Press, 1–272.

[ref47] Sullivan M. B. Moonaz S. Weber K. Taylor J. N. Schmalzl L. (2018). Toward an explanatory framework for yoga therapy informed by philosophical and ethical perspectives. Altern. Ther. Health Med. 24, 38–47. 29135457

[ref48] Venkataramiah M. (2006). Talks with Sri Ramana Maharshi: Three Volumes in One. Tiruvannamalai: Sri Ramanasramam.

[ref49] White D. G. (2012). Yoga in Practice. Princeton University Press.

[ref50] White D. G. (2014). The Yoga Sutra of Patanjali: A Biography, Lives of Great Religious Books. Princeton: Princeton University Press.

[ref51] Wittgenstein L. (1958). Philosophical Investigations. 2nd Edn New York: Macmillan.

[ref52] Yang S. Lu W. Yang D. Yao L. Wei B. (2015) “Short text understanding by leveraging knowledge into topic model,” in Proceedings of the 2015 Conference of the North American Chapter of the Association for Computational Linguistics: Human Language Technologies, eds. Mihalcea R. Chai J. Sarkar A. Denver: Association for Computational Linguistics 1232–1237

[ref53] Zhai Z. Liu B. Xu H. Jia P. (2011). “Constrained LDA for grouping product features in opinion mining,” in Advances in Knowledge Discovery and Data Mining, eds. Huang J. Z. Cao L. Srivastava J. (Berlin: Springer), 448–459.

