# Supplementary material for: Grammars of yoga: an exploratory text analytics approach to classical, pedagogical, scholarly, and scientific corpuses of yoga
Source: Front Psychol. 2026 Oct 26;17:1908435. doi: 10.3389/fpsyg.2026.1908435 (PMC13745233; doi:10.3389/fpsyg.2026.1908435)
Supplement: Supplementary file 1 [file Data_Sheet_1.DOCX]

Supplementary Material

# 1 Supplementary Data

## 1.1 Supplementary Figures

**Supplementary Figure 1.** Topic modeling analysis included developing a tokenized dataset (Supplementary Figure 1A) and a sensitivity analysis to examine and decide topic number (B).

**Supplementary Figure 1A.** Frequency distribution of word counts in the tokenized dataset. After splitting the text into sentences and sentences into words, extended stop words (e.g., yoga, etc.) were removed from each 200-word text block (also called documents). The histogram shows the number of words left in each document after cleaning, mapped against the number of documents with that number of words.

Number of Documents

Document word count

**Supplementary Figure 1B.** Three hyperparameters were simultaneously tuned to develop the number of topics. Below we show the (1) number of topics; (2) optimize_interval, which optimizes alpha (a prior belief on document-topic distribution) and beta (a prior belief on topic-word distribution) automatically; and (3) iterations: number of training iterations. Below are the values of hyperparameters that reached the highest topic coherence score for two, three, five, and six topics.

**Supplementary Figure 2:** Topic distribution and top-ranked terms from the unsupervised model (A) Topic distributions in the unsupervised model were generated using a pyLDAvis interactive plot. Data visualization revealed four topics with large, non-overlapping bubbles scattered throughout the chart, indicating a good topic model. In the resulting Inter-topic Distance Map, circles represent different topics and indicate the distance between each topic. Similar topics appear closer and dissimilar topics farther apart. The relative size of a topic circle in the plot corresponds to the relative frequency of the topic in the corpus. The larger the bubble, the higher the percentage of documents in the corpus related to that topic. Image shows setting λ=0. (B) t-SNE analysis confirms that the 200 documents form four clusters (blue= topic 1, yellow= topic 2, green= topic 3, red= topic 4). (C) The top terms in each of the four topics for the unsupervised model. Blue bars represent the overall frequency of each word in the corpus. Red bars give the estimated number of times a given term was generated by a given topic. The word with the longest red bar is the word that most used by documents belonging to that topic.

## 1.2 Supplementary Tables

**Supplementary Table 1A:** Attached to paper as a separate .csv file.

**Supplementary Table 1B: Characteristics of source types used for analysis**.

| **Source Type** | **Era** | **Date Range** | **Place of Composition** | **Number of Sources** |
| --- | --- | --- | --- | --- |
| **Sanskrit Texts** | Ancient-Modern | 6th century BCE-18th century CE | India | 48* |
| **Yoga Studies** | Contemporary | 1831-2025 CE | Australia, Austria, Canada, France, Germany, India, Israel, Italy, Korea, Mexico, Poland, Switzerland, UK, US | 50 |
| **Practice Manuals** | Modern-Contemporary | 1905-2020 CE | Canada, India, New Zealand, UK, US | 50 |
| **Scientific Research** | Contemporary | 2024-2026 CE | Australia, Brazil, China, Germany, Greece, Hong Kong, India, Italy, Norway, Sweden, Turkey, UAE, UK, US | 49** |

**Supplementary Table 2: Sensitivity analyses of the four topics.**

**Supplementary Table 2A** We tested different sets of anchor words to evaluate the stability of the results of topic modeling. We selected the top terms of each topic when setting λ=0.2, 0.4, 0.6, 0.8 and 1. This formed 5 alternative sets of anchor words. The main set of anchor words was selected from the top ranked terms when setting λ=0. The main and alternative sets of anchor words are shown below. Comparing these sets of anchor words, many different words show up when λ=0.2 versus λ=0. However, as λ increases, only new general terms are included and general terms become higher ranked instead of topic-specific terms. Using these alternative sets, we are able to test if the resulting topics are sensitive to the very different sets of anchor words and/or to similar sets of anchor words but with different order of within-set words.

λ=0

| Topic | Anchor Words |
| --- | --- |
| Topic 1 | [‘health’, ‘intervention’, ‘stress’, ‘breathing’, ‘improve’, ‘effect’, ‘benefit’, ‘treatment’, ‘relaxation’, ‘psychological’, ‘mental’, ‘balance’, ‘outcome’] |
| Topic 2 | [‘experience’, ‘union’, ‘consciousness’, ‘divine’, ‘realization’, ‘communion’, ‘universe’, ‘spirit’, ‘perfection’, ‘peace’, ‘state’, ‘harmony’, ‘power’] |
| Topic 3 | [‘text’, ‘century’, ‘philosophical’, ‘vedic’, ‘hindu’, ‘history’, ‘theory’, ‘jain’, ‘tradition’, ‘framework’, ‘logic’, ‘doctrine’] |
| Topic 4 | [‘free’, ‘sound’, ‘object’, ‘air’, ‘ātman’, ‘mantra’, ‘fire’, ‘pleasure’, ‘bond’, ‘praise’, ‘bliss’, ‘contemplation’] |

λ=0.2

| Topic | Anchor Words |
| --- | --- |
| Topic 1 | [‘health’, ‘mental’, ‘study’, ‘exercise’, ‘include’, ‘intervention’, ‘breathing’, ‘improve’, ‘effect’, ‘posture’, ‘benefit’, ‘stress’, ‘base’, ‘condition’] |
| Topic 2 | [‘body’, ‘state’, ‘life’, ‘nature’, ‘experience’, ‘spiritual’, ‘union’, ‘understand’, ‘science’, ‘human’, ‘consciousness’, ‘achieve’, ‘join’, ‘divine’] |
| Topic 3 | [‘term’, ‘discipline’, ‘tradition’, ‘century’, ‘text’, ‘modern’, ‘meaning’, ‘system’, ‘refer’, ‘religious’, ‘development’, ‘context’, ‘idea’, ‘practitioner’] |
| Topic 4 | [‘call’, ‘yogin’, ‘action’, ‘attain’, ‘object’, ‘knowledge’, ‘free’, ‘sound’, ‘practise’, ‘hold’, ‘sense’, ‘teach’, ‘limb’, ‘brahman’] |

λ=0.4

| Topic | Anchor Words |
| --- | --- |
| Topic 1 | [‘health’, ‘mental’, ‘study’, ‘exercise’, ‘physical’, ‘include’, ‘intervention’, ‘posture’, ‘breathing’, ‘improve’, ‘effect’, ‘stress’, ‘base’, ‘condition’] |
| Topic 2 | [‘body’, ‘mind’, ‘life’, ‘state’, ‘spiritual’, ‘nature’, ‘understand’, ‘experience’, ‘union’, ‘consciousness’, ‘science’, ‘human’, ‘achieve’, ‘divine’] |
| Topic 3 | [‘term’, ‘discipline’, ‘practice’, ‘tradition’, ‘system’, ‘century’, ‘modern’, ‘text’, ‘time’, ‘technique’, ‘practitioner’, ‘refer’, ‘meaning’, ‘word’] |
| Topic 4 | [‘call’, ‘yogin’, ‘action’, ‘attain’, ‘knowledge’, ‘object’, ‘free’, ‘sound’, ‘sense’, ‘teach’, ‘practise’, ‘brahman’, ‘begin’, ‘hold’] |

λ=0.6

| Topic | Anchor Words |
| --- | --- |
| Topic 1 | [‘health’, ‘mental’, ‘physical’, ‘study’, ‘exercise’, ‘include’, ‘posture’, ‘intervention’, ‘breathing’, ‘improve’, ‘effect’, ‘stress’, ‘base’, ‘meditation’] |
| Topic 2 | [‘body’, ‘mind’, ‘life’, ‘state’, ‘spiritual’, ‘understand’, ‘experience’, ‘nature’, ‘union’, ‘consciousness’, ‘practice’, ‘science’, ‘human’, ‘individual’] |
| Topic 3 | [‘practice’, ‘term’, ‘discipline’, ‘system’, ‘tradition’, ‘technique’, ‘time’, ‘modern’, ‘century’, ‘word’, ‘practitioner’, ‘text’, ‘refer’, ‘meaning’] |
| Topic 4 | [‘call’, ‘yogin’, ‘knowledge’, ‘action’, ‘attain’, ‘object’, ‘mind’, ‘free’, ‘sound’, ‘teach’, ‘sense’, ‘control’, ‘brahman’, ‘begin’] |

λ=0.8

| Topic | Anchor Words |
| --- | --- |
| Topic 1 | [‘physical’, ‘health’, ‘mental’, ‘study’, ‘exercise’, ‘include’, ‘practice’, ‘posture’, ‘intervention’, ‘meditation’, ‘breathing’, ‘improve’, ‘effect’, ‘stress’] |
| Topic 2 | [‘body’, ‘mind’, ‘life’, ‘practice’, ‘state’, ‘spiritual’, ‘understand’, ‘experience’, ‘nature’, ‘union’, ‘consciousness’, ‘science’, ‘individual’, ‘human’] |
| Topic 3 | [‘practice’, ‘term’, ‘system’, ‘technique’, ‘discipline’, ‘time’, ‘tradition’, ‘word’, ‘modern’, ‘practitioner’, ‘century’, ‘text’, ‘refer’, ‘yogic’] |
| Topic 4 | [‘call’, ‘mind’, ‘knowledge’, ‘yogin’, ‘action’, ‘attain’, ‘object’, ‘free’, ‘control’, ‘liberation’, ‘sound’, ‘teach’, ‘begin’, ‘sense’] |

λ=1

| Topic | Anchor Words |
| --- | --- |
| Topic 1 | [‘physical’, ‘health’, ‘mental’, ‘practice’, ‘study’, ‘exercise’, ‘include’, ‘posture’, ‘intervention’, ‘meditation’, ‘breathing’, ‘improve’, ‘effect’, ‘stress’] |
| Topic 2 | [‘body’, ‘mind’, ‘practice’, ‘life’, ‘state’, ‘spiritual’, ‘understand’, ‘experience’, ‘nature’, ‘union’, ‘consciousness’, ‘individual’, ‘science’, ‘human’] |
| Topic 3 | [‘practice’, ‘term’, ‘technique’, ‘system’, ‘discipline’, ‘time’, ‘word’, ‘tradition’, ‘practitioner’, ‘modern’, ‘century’, ‘text’, ‘yogic’, ‘refer’] |
| Topic 4 | [‘mind’, ‘call’, ‘knowledge’, ‘yogin’, ‘action’, ‘attain’, ‘control’, ‘object’, ‘free’, ‘liberation’, ‘breath’, ‘sound’, ‘teach’, ‘begin’] |

**Supplementary Table 2B** As sets of anchor words change, we need to re-tune the anchor-strength hyperparameter to find its optimal value to maximize the weighted average of specified evaluation metrics. Those hyperparameter values are presented below.

λ=0: anchor-strength=0.468444945512346

λ=0.2: anchor-strength= 0.351762551735987

λ=0.4: anchor-strength= 0.834826454577904

λ=0.6: anchor-strength= 0.948118021245789

λ=0.8: anchor-strength= 0.606793830054043

λ=1: anchor-strength= 0.542231553125466

**Supplementary Table 2C** We found that the top probable terms and their weights to each topic are similar across models with different sets of anchor words (namely models with different values of λ).

**Topic 1**

| **λ =0.2** | **λ =0.4** | **λ =0.6** | **λ =0.8** | **λ =1.0** |
| --- | --- | --- | --- | --- |
| Physical | Physical | Physical | Physical | Physical |
| Health | Health | Health | Practice | Health |
| Mental | Mental | Mental | Health | Practice |
| Practice | Study | Study | Mental | Mental |
| Exercise | Practice | Practice | Study | Exercise |
| Body | Exercise | Exercise | Exercise | Body |
| Study | Body | Body | Body | Study |
| Well | Include | Include | Include | Include |
| Include | Intervention | Well | Well | Well |
| Intervention | Well | Intervention | Intervention | Intervention |
| Breathing | Breathing | Breathing | Mind | Posture |
| Improve | Posture | Improve | Improve | Breathing |
| Stress | Improve | Stress | Breathing | Improve |
| Effect | Stress | Effect | Posture | Stress |
| Posture | Effect | Posture | Stress | Effect |
| Base | Base | Base | Effect | Base |
| Mind | Approach | Approach | Base | Control |
| Control | Mind | Condition | Approach | Condition |
| Condition | Condition | Activity | Condition | Activity |
| Reduce | Reduce | Reduce | Activity | Individual |

**Topic 2**

| **λ =0.2** | **λ =0.4** | **λ =0.6** | **λ =0.8** | **λ =1.0** |
| --- | --- | --- | --- | --- |
| Body | Mind | Body | Body | Body |
| Mind | Body | Mind | Mind | Mind |
| Mean | Mean | Mean | Mean | Mean |
| Practice | Self | Self | Self | Self |
| Self | Become | State | State | State |
| Word | Practice | Life | Way | Life |
| State | State | Practice | Life | Way |
| Way | Life | Way | Understand | Practice |
| Life | Spiritual | Spiritual | Practice | Understand |
| Understand | Way | Understand | Experience | Spiritual |
| Spiritual | Understand | Experience | Nature | Experience |
| Experience | Experience | Nature | High | Nature |
| Nature | Nature | Come | Come | Come |
| Come | Come | High | Spiritual | High |
| High | High | Union | Union | Union |
| Consciousness | Union | Consciousness | Consciousness | Consciousness |
| Union | Consciousness | Power | Sense | World |
| Power | Power | World | World | Power |
| World | World | Describe | Power | Physical |
| Describe | Sense | Soul | Describe | Know |

**Topic 3**

| **λ =0.2** | **λ =0.4** | **λ =0.6** | **λ =0.8** | **λ =1.0** |
| --- | --- | --- | --- | --- |
| Practice | Practice | Practice | Practice | Practice |
| System | Word | Word | Word | Word |
| Term | System | System | Different | System |
| Different | Different | Different | Term | Term |
| Use | Term | Term | Use | Different |
| Technique | Use | Use | Form | Use |
| Practitioner | Technique | Technique | Practitioner | Technique |
| Discipline | Meditation | Discipline | Discipline | Practitioner |
| Form | Discipline | Tradition | Technique | Discipline |
| Meditation | Form | Form | System | Form |
| Tradition | Tradition | Modern | Tradition | Tradition |
| Modern | Modern | Year | Meditation | Work |
| Time | Year | Time | Work | Modern |
| Year | Goal | Goal | Modern | Time |
| Ancient | Practitioner | Yogic | Time | Yogic |
| Yogic | Yogic | Practitioner | Year | Century |
| Century | Time | Century | Goal | Text |
| Various | Century | Meditation | Yogic | Part |
| Text | Text | Text | Century | Aspect |
| Part | Part | Method | Text | Philosophy |

**Topic 4**

| **λ=0.2** | **λ =0.4** | **λ =0.6** | **λ =0.8** | **λ =1.0** |
| --- | --- | --- | --- | --- |
| Mind | Call | Call | Mind | Mind |
| Call | Knowledge | Mind | Call | Knowledge |
| Knowledge | Yogin | Knowledge | Knowledge | Call |
| Say | Attain | Yogin | Become | Say |
| Become | Liberation | Say | Yogin | Yogin |
| Yogin | Breath | Attain | Attain | Become |
| Action | Say | Liberation | Liberation | Liberation |
| Liberation | Know | Become | Say | Attain |
| Attain | Object | Breath | Breath | Breath |
| Control | Free | Object | Object | Control |
| Breath | Well | Know | Concentration | Object |
| Object | Control | Free | Know | Concentration |
| Know | Great | Go | Free | Free |
| Concentration | Go | Teach | Control | Go |
| Give | Teach | Brahman | Go | Know |
| Free | Brahman | Control | Brahman | Teach |
| Begin | Union | Make | Action | Make |
| Go | Sense | Sense | Sense | Brahman |
| Teach | Make | Thus | Make | Sense |
| Brahman | Time | Form | Sound | Action |

**Supplementary Table 3: Regression estimates using predicted topic labels from (A) baseline LDAMallet Model and (B) GuidedLDA Model.**

To further examine the probability of topic assignment based on source type and the change in probability of topic assignment as source type changes, we used multinomial logistic regression. Supplementary Table 3A reports the estimates in both log odds ratio and odds ratio scales using the predicted topic labels from the baseline LDAMallet model. Supplementary 3B reports the equivalent using the predicted topic labels from the SeededLDA/GuidedLDA model.

(Supplementary Table 3A)

| Topic | Predictor | Estimate | SE | z_value | p_value | CI_lower | CI_upper | OR | OR_CI_lower | OR_CI_upper |
| --- | --- | --- | --- | --- | --- | --- | --- | --- | --- | --- |
| 2 | Intercept | 2.497 | 0.567 | 4.403 | 0 | 1.385 | 3.608 | 12.143 | 3.996 | 36.902 |
| 2 | Sanskrit Text | 0.336 | 1.589 | 0.212 | 0.832 | -2.777 | 3.45 | 1.4 | 0.062 | 31.511 |
| 2 | Scientific Research | -7.051 | 1.557 | -4.529 | 0 | -10.102 | -3.999 | 0.001 | 0 | 0.018 |
| 2 | Yoga Studies | -0.651 | 1.06 | -0.614 | 0.539 | -2.729 | 1.427 | 0.522 | 0.065 | 4.168 |
| 3 | Intercept | 0 | 0.771 | 0 | 1 | -1.511 | 1.511 | 1 | 0.221 | 4.531 |
| 3 | Sanskrit Text | 0 | 2.18 | 0 | 1 | -4.274 | 4.274 | 1 | 0.014 | 71.783 |
| 3 | Scientific Research | -2.608 | 0.956 | -2.729 | 0.006 | -4.481 | -0.735 | 0.074 | 0.011 | 0.48 |
| 3 | Yoga Studies | 3.219 | 1.147 | 2.807 | 0.005 | 0.971 | 5.467 | 25 | 2.641 | 236.696 |
| 4 | Intercept | -0.336 | 0.844 | -0.398 | 0.69 | -1.992 | 1.319 | 0.714 | 0.136 | 3.739 |
| 4 | Sanskrit Text | 4.779 | 1.679 | 2.847 | 0.004 | 1.489 | 8.069 | 119 | 4.433 | 3194.322 |
| 4 | Scientific Research | -4.217 | 1.678 | -2.514 | 0.012 | -7.506 | -0.929 | 0.015 | 0.001 | 0.395 |
| 4 | Yoga Studies | 1.184 | 1.305 | 0.907 | 0.364 | -1.374 | 3.742 | 3.267 | 0.253 | 42.183 |

(Supplementary Table 3B)

| Topic | Predictor | Estimate | SE | z_value | p_value | CI_lower | CI_upper | OR | OR_CI_lower | OR_CI_upper |
| --- | --- | --- | --- | --- | --- | --- | --- | --- | --- | --- |
| 2 | Intercept | 2.424 | 0.569 | 4.261 | 0 | 1.309 | 3.538 | 11.286 | 3.702 | 34.408 |
| 2 | Sanskrit Text | 0.285 | 1.594 | 0.178 | 0.858 | -2.841 | 3.41 | 1.329 | 0.058 | 30.252 |
| 2 | Scientific Research | -6.977 | 1.557 | -4.48 | 0 | -10.03 | -3.925 | 0.001 | 0 | 0.02 |
| 2 | Yoga Studies | 0.795 | 1.577 | 0.504 | 0.614 | -2.295 | 3.886 | 2.215 | 0.101 | 48.72 |
| 3 | Intercept | 0.251 | 0.727 | 0.346 | 0.73 | -1.173 | 1.676 | 1.286 | 0.309 | 5.343 |
| 3 | Sanskrit Text | -0.251 | 2.165 | -0.116 | 0.908 | -4.495 | 3.993 | 0.778 | 0.011 | 54.193 |
| 3 | Scientific Research | -2.859 | 0.92 | -3.106 | 0.002 | -4.663 | -1.055 | 0.057 | 0.009 | 0.348 |
| 3 | Yoga Studies | 4.066 | 1.624 | 2.504 | 0.012 | 0.884 | 7.248 | 58.333 | 2.42 | 1405.819 |
| 4 | Intercept | 0.251 | 0.727 | 0.346 | 0.73 | -1.173 | 1.676 | 1.286 | 0.309 | 5.343 |
| 4 | Sanskrit Text | 4.215 | 1.622 | 2.598 | 0.009 | 1.035 | 7.394 | 67.667 | 2.814 | 1626.988 |
| 4 | Scientific Research | -4.805 | 1.622 | -2.963 | 0.003 | -7.984 | -1.627 | 0.008 | 0 | 0.197 |
| 4 | Yoga Studies | 0.847 | 1.817 | 0.466 | 0.641 | -2.714 | 4.409 | 2.333 | 0.066 | 82.16 |

**Supplementary Table 4. Average predicted probabilities of topics by source type using predicted topic labels from the (A) baseline LDAMallet model (B) GuidedLDA Model.**

We also derived the average predicted probabilities from the penalized multinomial logistic model to better understand the model. We derived the average predicted probability of being assigned to each topic at each source type of yoga definition. Supplementary Table 4A reports those average predicted probabilities using the predicted topic labels from the baseline LDAMallet model. Supplementary Table 4B reports the equivalent using the predicted topic labels from the SeededLDA/GuidedLDA model.

Supplementary Table 4A shows that the average predicted probability of being assigned to topic 1 when the source type is scientific research is 0.91346 (95% CI: [0.83553, 0.9914]). The average predicted probability of being assigned to topic 2 when the source type is practice manual is 0.81731 (95% CI: [0.71020 0.9244]). The average predicted probability of being assigned to topic 3 when the source type is yoga studies is 0.72115 (95% CI: [0.59686 0.8455]). And the average predicted probability of being assigned to topic 4 when the source type is Sanskrit text is 0.81731 (95% CI: [0.71020 0.9244]). Supplementary Table 4B shows that the average predicted probability of being assigned to topic 1 when the source type is scientific research is 0.91346 (95% CI: [0.83553, 0.9914]). The average predicted probability of being assigned to topic 2 when the source type is practice manual is 0.75962 (95% CI: [0.64117 0.8781]). The average predicted probability of being assigned to topic 3 when the source type is yoga studies is 0.72115 (95% CI: [0.59686 0.8455]). And the average predicted probability of being assigned to topic 4 when the source type is Sanskrit text is 0.83654 (95% CI: [0.73404 0.9390]).

(Supplementary Table 4A)

| Topic | Source Type | estimate | std.error | z | p | conf.low | conf.high |
| --- | --- | --- | --- | --- | --- | --- | --- |
| 1 | Practice Manual | 0.06731 | 0.0354 | 1.899 | 0.05750 | -0.00214 | 0.1368 |
| 1 | Sanskrit Text | 0.00962 | 0.0138 | 0.697 | 0.48597 | -0.01743 | 0.0367 |
| 1 | Scientific Research | 0.91346 | 0.0398 | 22.973 | 8.602e-117 | 0.83553 | 0.9914 |
| 1 | Yoga Studies | 0.02885 | 0.0237 | 1.219 | 0.22297 | -0.01755 | 0.0752 |
| 2 | Practice Manual | 0.81731 | 0.0546 | 14.956 | 1.422e-50 | 0.71020 | 0.9244 |
| 2 | Sanskrit Text | 0.16346 | 0.0523 | 3.126 | 0.00177 | 0.06096 | 0.2660 |
| 2 | Scientific Research | 0.00962 | 0.0138 | 0.697 | 0.48597 | -0.01743 | 0.0367 |
| 2 | Yoga Studies | 0.18269 | 0.0546 | 3.343 | 0.00083 | 0.07559 | 0.2898 |
| 3 | Practice Manual | 0.06731 | 0.03543 | 1.900 | 0.05750 | -0.00214 | 0.1368 |
| 3 | Sanskrit Text | 0.00962 | 0.01380 | 0.697 | 0.48597 | -0.01743 | 0.0367 |
| 3 | Scientific Research | 0.06731 | 0.03543 | 1.900 | 0.05749 | -0.00214 | 0.1368 |
| 3 | Yoga Studies | 0.72115 | 0.06342 | 11.371 | 5.800e-30 | 0.59686 | 0.8455 |
| 4 | Practice Manual | 0.04808 | 0.03025 | 1.589 | 0.11204 | -0.01122 | 0.1074 |
| 4 | Sanskrit Text | 0.81731 | 0.05465 | 14.956 | 1.422e-50 | 0.71020 | 0.9244 |
| 4 | Scientific Research | 0.00962 | 0.01380 | 0.697 | 0.48597 | -0.01743 | 0.0367 |
| 4 | Yoga Studies | 0.06731 | 0.03543 | 1.900 | 0.05749 | -0.00214 | 0.1368 |

(Supplementary Table 4B)

| Topic | Source.Type | estimate | std.error | statistic | p.value | conf.low | conf.high |
| --- | --- | --- | --- | --- | --- | --- | --- |
| 1 | Practice Manual | 0.0673 | 0.0354 | 1.8995 | 0.0575 | -0.0021 | 0.1368 |
| 1 | Sanskrit Text | 0.0096 | 0.0138 | 0.6967 | 0.4860 | -0.0174 | 0.0367 |
| 1 | Scientific Research | 0.9135 | 0.0398 | 22.9734 | 0.0000 | 0.8355 | 0.9914 |
| 1 | Yoga Studies | 0.0096 | 0.0138 | 0.6967 | 0.4860 | -0.0174 | 0.0367 |
| 2 | Practice Manual | 0.7596 | 0.0604 | 12.5698 | 0.0000 | 0.6412 | 0.8781 |
| 2 | Sanskrit Text | 0.1442 | 0.0497 | 2.9029 | 0.0037 | 0.0469 | 0.2416 |
| 2 | Scientific Research | 0.0096 | 0.0138 | 0.6967 | 0.4860 | -0.0174 | 0.0367 |
| 2 | Yoga Studies | 0.2404 | 0.0604 | 3.9778 | 0.0001 | 0.1219 | 0.3588 |
| 3 | Practice Manual | 0.0865 | 0.0398 | 2.1764 | 0.0295 | 0.0086 | 0.1645 |
| 3 | Sanskrit Text | 0.0096 | 0.0138 | 0.6967 | 0.4860 | -0.0174 | 0.0367 |
| 3 | Scientific Research | 0.0673 | 0.0354 | 1.8995 | 0.0575 | -0.0021 | 0.1368 |
| 3 | Yoga Studies | 0.7212 | 0.0634 | 11.3715 | 0.0000 | 0.5969 | 0.8455 |
| 4 | Practice Manual | 0.0865 | 0.0398 | 2.1764 | 0.0295 | 0.0086 | 0.1645 |
| 4 | Sanskrit Text | 0.8365 | 0.0523 | 15.9963 | 0.0000 | 0.7340 | 0.9390 |
| 4 | Scientific Research | 0.0096 | 0.0138 | 0.6967 | 0.4860 | -0.0174 | 0.0367 |
| 4 | Yoga Studies | 0.0288 | 0.0237 | 1.2187 | 0.2230 | -0.0175 | 0.0752 |

**Supplementary Table 5 Average Marginal Effects of Source Type Using Predicted Topic Labels from (A) Baseline LDAMallet and (B) GuidedLDA Model**

We further derived the average marginal effects of source type on the predicted probability of being assigned to each topic rather than to all other topics from Supplementary Table 4A and Supplementary Table 4B, as reported below in Supplementary Table 5A and Supplementary Table 5B, respectively. In Supplementary Table 5A, as source type changes from practice manual to Sanskrit text, the probability of being assigned to topic 1 rather than all other topics decreases by 0.0577. As source type changes from practice manual to scientific research, the probability of being assigned to topic 1 rather than all other topics increases by 0.846. As source type changes from practice manual to yoga studies, the probability of being assigned to topic 1 rather than all other topics decreases by 0.0385. As source type changes from practice manual to Sanskrit text, the probability of being assigned to topic 2 rather than all other topics decreases by 0.654. As source type changes from practice manual to scientific research, the probability of being assigned to topic 2 rather than all other topics decreases by 0.808. As source type changes from practice manual to yoga studies, the probability of being assigned to topic 2 rather than all other topics decreases by 0.635. As source type changes from practice manual to Sanskrit text, the probability of being assigned to topic 3 rather than all other topics decreases by 0.0577. As source type changes from practice manual to scientific research, the probability of being assigned to topic 3 rather than all other topics has little change. As source type changes from practice manual to yoga studies, the probability of being assigned to topic 3 rather than all other topics increases by 0.654. As source type changes from practice manual to Sanskrit text, the probability of being assigned to topic 4 rather than all other topics increases by 0.769. As source type changes from practice manual to scientific research, the probability of being assigned to topic 4 rather than all other topics decreases by 0.0385. As source type changes from practice manual to yoga studies, the probability of being assigned to topic 4 rather than all other topics increases by 0.0192.

Supplementary Table 5B can be interpreted through the same process.

(Supplementary Table 5A)

| Topic | contrast | estimate | std.error | z | p | conf.low | conf.high |
| --- | --- | --- | --- | --- | --- | --- | --- |
| 1 | Sanskrit Text – Practice Manual | -0.0577 | 0.0380 | -1.5172 | 0.1292 | -0.1322 | 0.0168 |
| 1 | Scientific Research – Practice Manual | 0.8462 | 0.0533 | 15.8876 | 0.0000 | 0.7418 | 0.9505 |
| 1 | Yoga Studies – Practice Manual | -0.0385 | 0.0426 | -0.9026 | 0.3667 | -0.1220 | 0.0451 |
| 2 | Sanskrit Text – Practice Manual | -0.6538 | 0.0756 | -8.6444 | 0.0000 | -0.8021 | -0.5056 |
| 2 | Scientific Research – Practice Manual | -0.8077 | 0.0564 | -14.3302 | 0.0000 | -0.9182 | -0.6972 |
| 2 | Yoga Studies – Practice Manual | -0.6346 | 0.0773 | -8.2116 | 0.0000 | -0.7861 | -0.4831 |
| 3 | Sanskrit Text – Practice Manual | -0.0577 | 0.0380 | -1.5172 | 0.1292 | -0.1322 | 0.0168 |
| 3 | Scientific Research – Practice Manual | 0.0000 | 0.0501 | 0.0000 | 1.0000 | -0.0982 | 0.0982 |
| 3 | Yoga Studies – Practice Manual | 0.6538 | 0.0726 | 9.0005 | 0.0000 | 0.5115 | 0.7962 |
| 4 | Sanskrit Text – Practice Manual | 0.7692 | 0.0625 | 12.3150 | 0.0000 | 0.6468 | 0.8917 |
| 4 | Scientific Research – Practice Manual | -0.0385 | 0.0333 | -1.1566 | 0.2474 | -0.1036 | 0.0267 |
| 4 | Yoga Studies – Practice Manual | 0.0192 | 0.0466 | 0.4127 | 0.6798 | -0.0721 | 0.1106 |

(Supplementary Table 5B)

| Topic | contrast | estimate | std.error | z | p.value | conf.low | conf.high |
| --- | --- | --- | --- | --- | --- | --- | --- |
| 1 | Sanskrit Text – Practice Manual | -0.0577 | 0.0380 | -1.5172 | 0.1292 | -0.1322 | 0.0168 |
| 1 | Scientific Research – Practice Manual | 0.8462 | 0.0533 | 15.8875 | 0.0000 | 0.7418 | 0.9505 |
| 1 | Yoga Studies – Practice Manual | -0.0577 | 0.0380 | -1.5172 | 0.1292 | -0.1322 | 0.0168 |
| 2 | Sanskrit Text – Practice Manual | -0.6154 | 0.0782 | -7.8659 | 0.0000 | -0.7687 | -0.4620 |
| 2 | Scientific Research – Practice Manual | -0.7500 | 0.0620 | -12.0992 | 0.0000 | -0.8715 | -0.6285 |
| 2 | Yoga Studies – Practice Manual | -0.5192 | 0.0855 | -6.0755 | 0.0000 | -0.6867 | -0.3517 |
| 3 | Sanskrit Text – Practice Manual | -0.0769 | 0.0421 | -1.8276 | 0.0676 | -0.1594 | 0.0056 |
| 3 | Scientific Research – Practice Manual | -0.0192 | 0.0533 | -0.3611 | 0.7180 | -0.1236 | 0.0852 |
| 3 | Yoga Studies – Practice Manual | 0.6346 | 0.0749 | 8.4783 | 0.0000 | 0.4879 | 0.7813 |
| 4 | Sanskrit Text – Practice Manual | 0.7500 | 0.0657 | 11.4164 | 0.0000 | 0.6212 | 0.8788 |
| 4 | Scientific Research – Practice Manual | -0.0769 | 0.0421 | -1.8276 | 0.0676 | -0.1594 | 0.0056 |
| 4 | Yoga Studies – Practice Manual | -0.0577 | 0.0463 | -1.2468 | 0.2125 | -0.1484 | 0.0330 |

**Supplementary Table 6. IIA Test Using Predicted Topic Labels from (A) baseline LDAMallet and (B) GuidedLDA Model**

The odds ratio of outcome m versus n is determined without reference to the other outcomes that might be available. This is the IIA assumption of multinomial logistic regression. We tested this assumption informally by dropping each outcome category, refitting the model, and checking whether coefficients for the remaining outcome categories change. IIA likely holds with small changes. It is likely violated when there are large systematic changes. Supplementary Table 6A reports the test results using predicted topic labels from the baseline LDAMallet model. Supplementary Table 6B reports the equivalent using predicted topic label from SeededLDA/GuidedLDA model.

When dropping topic 2, the odds ratios of topic 3 vs. topic 1 and topic 4 vs. topic 1 were unchanged. The same occurred when dropping topic 3 or topic 4. When dropping topic 1, the odds ratios of topic 3 vs. topic 2 and topic 4 vs. topic 2 changed substantially. But these large coefficient differences are not the violation of IIA because in multinomial logit, coefficients are defined relative to a reference (baseline) outcome category. When dropping a topic, the baseline can change and the entire coefficient system is reparametrized. Even if IIA holds, coefficients can change dramatically due to baseline shift effect. Based on the evidence in the situation where the baseline outcome category (topic 1) keeps the same across contrasting models when dropping alternative outcomes (topic 2, 3, or 4), the IIA assumption holds.

(Supplementary Table 6A)

| Dropped_Topic | Topic | Predictor | Coef_Diff |
| --- | --- | --- | --- |
| 1 | 3 | Intercept | -2.496741107 |
| 1 | 3 | Sanskrit Text | -0.336472237 |
| 1 | 3 | Scientific Research | 7.050617999 |
| 1 | 3 | Yoga Studies | 0.650914417 |
| 1 | 4 | Intercept | -2.496741107 |
| 1 | 4 | Sanskrit Text | -0.336472237 |
| 1 | 4 | Scientific Research | 7.050617999 |
| 1 | 4 | Yoga Studies | 0.650914417 |
| 2 | 3 | Intercept | -1.29E-15 |
| 2 | 3 | Sanskrit Text | 2.28E-14 |
| 2 | 3 | Scientific Research | 4.44E-16 |
| 2 | 3 | Yoga Studies | 3.55E-15 |
| 2 | 4 | Intercept | -3.16E-15 |
| 2 | 4 | Sanskrit Text | 2.04E-14 |
| 2 | 4 | Scientific Research | 6.22E-15 |
| 2 | 4 | Yoga Studies | 5.33E-15 |
| 3 | 2 | Intercept | 8.88E-16 |
| 3 | 2 | Sanskrit Text | 5.66E-15 |
| 3 | 2 | Scientific Research | 3.55E-15 |
| 3 | 2 | Yoga Studies | -2.78E-15 |
| 3 | 4 | Intercept | 7.22E-16 |
| 3 | 4 | Sanskrit Text | 5.33E-15 |
| 3 | 4 | Scientific Research | 4.44E-15 |
| 3 | 4 | Yoga Studies | -2.00E-15 |
| 4 | 2 | Intercept | 2.66E-15 |
| 4 | 2 | Sanskrit Text | 8.10E-15 |
| 4 | 2 | Scientific Research | -2.66E-15 |
| 4 | 2 | Yoga Studies | -2.66E-15 |
| 4 | 3 | Intercept | 3.33E-15 |
| 4 | 3 | Sanskrit Text | 1.55E-14 |
| 4 | 3 | Scientific Research | -4.44E-15 |
| 4 | 3 | Yoga Studies | -3.55E-15 |

(Supplementary Table 6B)

| Dropped_Topic | Topic | Predictor | Coef_Diff |
| --- | --- | --- | --- |
| 1 | 3 | Intercept | -2.423537703 |
| 1 | 3 | Sanskrit Text | -0.284512498 |
| 1 | 3 | Scientific Research | 6.977414595 |
| 1 | 3 | Yoga Studies | -0.795338121 |
| 1 | 4 | Intercept | -2.423537703 |
| 1 | 4 | Sanskrit Text | -0.284512498 |
| 1 | 4 | Scientific Research | 6.977414595 |
| 1 | 4 | Yoga Studies | -0.795338121 |
| 2 | 3 | Intercept | 2.78E-15 |
| 2 | 3 | Sanskrit Text | 1.22E-15 |
| 2 | 3 | Scientific Research | -3.11E-15 |
| 2 | 3 | Yoga Studies | -8.88E-16 |
| 2 | 4 | Intercept | 3.83E-15 |
| 2 | 4 | Sanskrit Text | -3.55E-15 |
| 2 | 4 | Scientific Research | -5.33E-15 |
| 2 | 4 | Yoga Studies | -1.22E-15 |
| 3 | 2 | Intercept | 2.66E-15 |
| 3 | 2 | Sanskrit Text | -3.33E-15 |
| 3 | 2 | Scientific Research | -7.99E-15 |
| 3 | 2 | Yoga Studies | -7.44E-15 |
| 3 | 4 | Intercept | 3.55E-15 |
| 3 | 4 | Sanskrit Text | -3.55E-15 |
| 3 | 4 | Scientific Research | -4.44E-15 |
| 3 | 4 | Yoga Studies | -7.22E-15 |
| 4 | 2 | Intercept | 3.55E-15 |
| 4 | 2 | Sanskrit Text | -4.11E-15 |
| 4 | 2 | Scientific Research | -4.44E-15 |
| 4 | 2 | Yoga Studies | -9.99E-16 |
| 4 | 3 | Intercept | 3.22E-15 |
| 4 | 3 | Sanskrit Text | -3.72E-15 |
| 4 | 3 | Scientific Research | -4.00E-15 |
| 4 | 3 | Yoga Studies | -1.78E-15 |

**Supplementary Table 7. Top texts in each topic**

**Topic 1 (dominated by scientific research)**

1. Oliviera et al The effect of an Iyengar yoga-based exercise programme versus a seated yoga relaxation programme on falls in people aged 60 years and older (SAGE): a pragmatic, two-arm, parallel randomised controlled trial

2. Hough et al Effects of Hatha Yoga vs Physical Conditioning on Sleep in Women With Urinary Incontinence: A Secondary Analysis of a Randomized Clinical Trial.

3. Sujan et al Yoga-based breathing and relaxation as adjunctive therapy for chronic migraine: A randomized controlled trial on clinical outcomes and autonomic regulation

4. Girdharan Effects of Yoga Interventions on Health Outcomes in Women With Gynecological Cancers: A Systematic Review of Randomised Controlled Trials.

5. Pan et al Meta-analysis of yoga-based mind-body interventions in the management of inflammatory bowel disease

6. Durusoy et al The role of yoga as mind-body exercise in fibromyalgia management: A systematic review

7. Feldman et al Therapeutic relationship in group cognitive behavior therapy, yoga, and stress education for generalized anxiety disorder.

8. Pasarica et al Yoga for Wellness: An Innovative Educational Intervention for Health Professions Students.
9. Sharma et al. Effect of Yoga-based Interventions on Heart Rate Variability and Anxiety Among Students: A Systematic Review and Meta-analysis.
10. Li et al Analysis of risk factors affecting knee injury during yoga based on medical big data analysis.

**Topic 2 (dominated by practice manuals)**

1. Amaladass Christian Responses to Yoga in the Second Half of the Twentieth Century

2. Christensen The American Yoga Association Beginner's Manual Fully Revised and Updated.

3. Keller Hatha Yoga in the Anusara Style

4. Wittich Immortality and Longevity through Yoga in the Tirumantiram

5. *Parākhyatantra*

6. Swami Jyotir Maya Nanda Jnana Yoga: Secrets of Wisdom

7. Siri Singh Sahib Bhai Sahib and Yogi Bhajan Sadhana Guidelines for Kundalini Yoga Daily Practice

8. Paramahansa Yogananda God Talks With Arjuna The Bhagavad Gita Royal Science Of God Realization

9. Glen Alexander Eroticism and Cosmic Transformation as Yoga: The Ātmatattva of the Vaisnava Sahajiyās of Bengal

10. Keshub Chandra Sen's Lectures in India

**Topic 3 (dominated by yoga studies)**

1. Christopher Jain: Engaged Alchemies: New Approaches to the Study of Contemporary Yoga
2. Kengo Harimoto How Yoga Became Yoga
3. Dolly Yang Knowledge Transfer of Bodily Practices Between China and India in the Mediaeval World
4. Beatrix Hauser The health imaginary of postural yoga
5. Ian Baker Tibetan Yoga: Somatic Practice in Vajrayāna Buddhism and Dzogchen
6. Rocco Cestola Fading into Death through Pātañjalayoga: On the Apparent Death-Like State of the Yoga Practitioner Absorbed into Contentless Samādhi.
7. Farah Godrej The Neoliberal Yogi and the Politics of Yoga
8. Stuart Sarbacker Tracing the path of Yoga: the history and philosophy of Indian mind-body discipline
9. Samani Pratibha Pragya Yoga and Meditation in the Jain Tradition
10. Theodora Wildcroft Patterns of authority and practice relationships in ‘post-lineage yoga’

**Topic 4 (dominated by Sanskrit texts)**

1. *Ṛg Veda I.34*

2. *Maṇḍalabrahmopaniṣad*

3. *Ṛg Veda I.19*

4. *Brahmavidyopaniṣad*

5. *Paramārthasaṃgraha*

6. *Khecarīvidyā*

7. *Yoga Sūtra of Patañjali*

8. *Amanaska*

9. *Amṛtaṇopaniṣad*

10. *Darśanopaniṣad*

**Supplementary Table 8.** Texts that cluster atypically/ sources that are in topics distant from their source type

| **Non-scientific research sources in topic 1** | | | |
| --- | --- | --- | --- |
| **Author** | **Text** | **Source Type** | **Topic Perc Contribution** |
| Indra Devi | Yoga for Americans | Practice Manual | 0.4897 |
| Mark Stephens | Yoga Sequencing | Practice Manual | 0.3941 |
| Kino MacGregor | The Power of Ashtanga Yoga: Developing a Practice That Will Bring You Strength, Flexibility, and Inner Peace. | Practice Manual | 0.3547 |

| **Non-practice manual sources in topic 2** | | |  |
| --- | --- | --- | --- |
| **Author** | **Text** | **Source Type** | **Topic Perc Contribution** |
|  | *Parākhyatantra* | Sanskrit Text | 0.7277 |
|  | *Yoga Cudamani Upaniṣad* | Sanskrit Text | 0.6235 |
|  | Kaṭha Upaniṣasd | Sanskrit Text | 0.5677 |
| Vyāsa/Patañjali | *Yoga Sūtra Bhaṣya* | Sanskrit Text | 0.5511 |
| Hariharānanda Araṇya | *Kāpilāśramīya-Stotrasāmgrahah* | Sanskrit Text (modern) | 0.5294 |
|  | *Yogatattva Upaniṣad* | Sanskrit Text | 0.509 |
| Kauṇḍinya | Kauṇḍinya Commentary on the Paśupata Sūtra | Sanskrit Text | 0.4352 |
| Anand Amaladass | Christian Responses to Yoga in the Second Half of the Twentieth Century | Yoga Studies | 0.7954 |
| Agi Wittich | Immortality and Longevity through Yoga in the Tirumantiram | Yoga Studies | 0.7432 |
| Glen Alexander Hayes | Eroticism and Cosmic Transformation as Yoga: The Ātmatattva of the Vaisnava Sahajiyās of Bengal | Yoga Studies | 0.6929 |
| Keshub Chandra Sen | Keshub Chandra Sen's Lectures in India | Yoga Studies | 0.6923 |
| Joseph Alter | Yoga, Nature Cure and “Perfect” Health: The Purity of the Fluid Body in an Impure World | Yoga Studies | 0.5861 |
| Christele Barois | The Dharmaputrikā Saṃhitā: Preliminary Notes on an Early Text on Yoga | Yoga Studies | 0.463 |
| Marcel Mauss | Techniques of the Body | Yoga Studies | 0.4447 |
| Karl-Stéphan Bouthillette | What is Yogic Gnosticism? Demarcating an Elite Ritual Identity Worth Reconsidering | Yoga Studies | 0.4446 |
| Dominic Wujastyk | The Path to Liberation through Yogic Mindfulness in early Āyurveda | Yoga Studies | 0.4224 |
| Daniel Simpson | The Truth of Yoga: A Comprehensive Guide to Yoga's History, Texts, Philosophy, and Practices. | Yoga Studies | 0.4138 |
| Finnian M Gererty | Yoga and Sound | Yoga Studies | 0.3982 |
| Richard Davis | The Bhagavad Gita: A Biography | Yoga Studies | 0.3831 |

| **Non-yoga studies sources in topic 3** | | | |
| --- | --- | --- | --- |
| **Author** | **Text** | **Source Type** | **Topic Perc Contribution** |
| TKV Desikachar and R. H. Cravens. | Health, Healing, and Beyond: Yoga and the Living Tradition of Krishnamacharya | Practice Manual | 0.5132 |
| Gary Kraftsow | Yoga for Wellness | Practice Manual | 0.4541 |
| Marlysa Sullivan | Yoga Therapy: Applied Philosophy and Science for Health and Well-Being. | Practice Manual | 0.4113 |
| Radhanatha Swami | The Journey Within | Practice Manual | 0.3876 |
| Ghai et al. | Effects of Yoga Nidra on Stress, Anxiety, and Depression: A Systematic Review and Meta-Analysis. | Scientific paper | 0.5234 |
| Chetry et al. | A Comparison of the Effects of Three Volitional Yoga Breathing Practices versus Two Nonvolitional Breathing Controls on Auditory-evoked Potentials. | Scientific paper | 0.5139 |
| Malone et al. | A Black feminist grounded theory of embodiment through yoga among Black womxn | Scientific paper (one of the few qualitative studies) | 0.5115 |

| **Non-Sanskrit text sources in topic 4** | | | |
| --- | --- | --- | --- |
| **Author** | **Text** | **Source Type** | **Topic Perc Contribution** |
| Venkataramiah Munagala | Talks with Sri Ramana Maharshi | Practice Manual | 0.5087 |
| Tirumalai Krishnamacarya | Yoga Makaranda | Practice Manual | 0.4715 |
| A.C. Bhaktivedanta Swami Prabhupāda | The Perfection of Yoga | Practice Manual | 0.4562 |
| Bikram Choudhary | Bikram's Beginning Yoga Class | Practice Manual | 0.3948 |
| Sthaneshwar Timalsina | The Yoga System of the Josmanīs | Yoga Studies | 0.4088 |

## 1.3 Supplementary Methods

1.3.1 The LDAMalletModel

The Mallet module allows LDA model estimation using an (optimized version of) collapsed gibbs sampling from MALLET, an efficient method for document-topic hyperparameter optimization. To use it requires installing a specific version of GENSIM package and setting up os.environ[‘JAVA_HOME’], os.environ[‘MALLET_HOME’] and mallet_path variables.

1.3.2 Hyperparameter Tuning to Decide the Optimal Number of Topics

Next we used hyperparameter tuning to decide the optimal number of topics using three hyperparameters: (1) number of topics; (2) optimize_interval, which optimizes alpha (i.e., prior belief on document-topic distribution) and beta (prior belief on topic-word distribution) automatically, and (3) iterations: number of training iterations.

topics_range = range(2, 20, 1)

OptimizeIntervals = [10, 50, 100, 300, 500, 1000, 2000, 3000, 7000]

NumIterations = [3000, 6000]

We used two validation sets to test the consistency of results: 75% corpus and 100% corpus. We used topic coherence score as the criterion to select the optimal combination of these three hyperparameters. We selected the combination of the three hyperparameters that reached the highest topic coherence score: num_topics=4, optimize_interval=500, iterations=3000. The final model used was the Lda Mallet Model with these hyperparameter values.

We used two validation sets to test the consistency of results: 75% corpus and 100% corpus. We used topic coherence score as the criterion to select the optimal combination of these three hyperparameters. We selected the combination of the three hyperparameters that reached the highest topic coherence score: num_topics=4, optimize_interval=500, iterations=3000. The final model used was the Lda Mallet Model with these hyperparameter values.

1.3.3 Semi-supervised topic modeling

Since the unsupervised LDA model can be prone to creating topics that are not meaningful (Chang et al., 2009). Semi-supervised topic models allow users to apply their domain knowledge to nudge the model results in the desired direction (Andrzejewski et al., 2009; Andrzejewski and Zhu, 2009; Gallagher et al., 2017; Jagarlamudi et al., 2012; Yang et al., 2015; Zhai et al., 2011). Since the most probable terms for topics from the ldaMallet model were sometimes too generic, we used SeededLDA/GuidedLDA model to form four more coherent topics. SeededLDA/GuidedLDA (Jagarlamudi et al., 2012) is a semi-supervised LDA extension. This approach allowed us to provide sets of anchor words representative of the corpus to guide the topic discovery process. In our study, anchor words are used for all hidden topics. Two aspects of LDA’s generative process were modified to infuse the topic model with the user’s domain knowledge. First, topic-word proportions were upgraded using anchor words. Second, anchor sets were used to improve the document-topic distribution.

1.3.4 Domain Knowledge

In semi-supervised topic modeling, domain knowledge needs to be supplied to the algorithms in the form of lists of words that are representative of certain topics. These words are called "anchor words". We provided anchor words to guide the topic modeling results toward topics that cohered around specific themes. To achieve this, we used the pyLDAvis tool to obtain the top relevant terms ranked by their exclusiveness to a topic from the LdaMallet model (Table 2). These terms are the most distinctive/unique words of the topic that make the topic special but may include rare or less interpretable words. We selected words from them and added our own based on our domain knowledge to create our anchor words for this study. The anchor words are provided in Table 1.

1.3.5 Outputs/Results

The SeededLDA/GuidedLDA model produces outputs very similar to standard LDA, with a few additions due to its semi-supervised nature. After fitting a GuidedLDA model, using model.topic_word_ attribute, we obtained the topic-word distribution, where words are ranked by their probability within a topic and anchor words get a higher probability in the assigned topics. Using the model.transform() function over the document-term matrix of our dataset, we obtained the topic distribution for each document. From this, we got the dominant topic per document.

1.3.6 Hyperparameter Tuning

We did not tune the number of topics for the guided model since we knew from the ldaMallet model that there should be four topics in the corpus. Given four topics, we first tuned the hyperparameters alpha (a prior belief on document-topic distribution) and beta (a prior belief on topic-word distribution) to maximize the weighted average of topic coherence and topic diversity of the model. The GuidedLDA package we used allows us to set the strength of the domain knowledge 0 ≤ s ≤ 1, which will impact whether anchored topics or regular topics are used to generate words. Thus, we looked for the optimal value of s to maximize the weighted average of topic coherence, seed f1 score, seed usage score, and topic diversity of the model as well. We tuned alpha and beta first and then tuned s given the chosen alpha and beta values. We tuned the three hyperparameters—alpha, beta, and s—sequentially instead of simultaneously because only sequential tuning guarantees the correct mapping of a topic and its associated anchor words. The order of topics changes as alpha and beta change. Once alpha and beta was fixed, the order of topics was fixed, providing anchored words matching that order of topics. Anchor strength s will not change the order of topics, maintaining the correct mapping.

With four topics, alpha=0.677, beta=0.0048 maximize the weighted average of topic coherence and topic diversity of the model. Given four topics and these alpha and beta values, anchor strength s=0.468 maximizes the weighted average of topic coherence, seed f1 score, seed usage score, and topic diversity of the model. Interestingly, the grid search for SeededLDA suggests the use of only modest domain knowledge strength.

1.3.7 Summary/Descriptive Statistics

Tables 3 shows the topic distribution by source type of yoga definition in counts from the SeededLDA/GuidedLDA model, respectively. Across the two models an overwhelmingly large proportion of the yoga definitions from scientific research talks about topic 1; an overwhelmingly large proportion of the yoga definitions from practice manual talks about topic 2; an overwhelmingly large proportion of the yoga definitions from yoga studies talks about topic 3; and an overwhelmingly large proportion of the yoga definitions from Sanskrit text talks about topic 4. To further know if this association between topic label and source type is statistically significant, we performed statistical testing and modeling.

1.3.8 Statistical Analysis

*Chi-square test of independence*

We got chi-squared = 383.72, df = 9, p-value < 2.2e-16 for the baseline ldaMallet model and chi-squared = 377.87, df = 9, p-value < 2.2e-16 for the SeededLDA/GuidedLDA model. We thus rejected the null hypothesis and concluded that the topic assignment was significantly associated with the source type of yoga definition. One caveat is that the Chi-square test assumes cell counts ≥ 5 (rough guideline). We have some cell counts zeros. But the results are still valid here because the signal is very strong. After this test, we further plotted the standardized residuals, which gave us which topic-source type pairs were driving the association. Figure 4 show the topic dominance by source type via Chi-square residuals from the baseline ldaMallet model and the SeededLDA/GuidedLDA model, respectively. We got chi-squared = 383.72, df = 9, p-value < 2.2e-16 for the baseline ldaMallet model and chi-squared = 377.87, df = 9, p-value < 2.2e-16 for the SeededLDA/GuidedLDA model. We thus rejected the null hypothesis and concluded that the topic assignment was significantly associated with the source type of yoga definition. Standardized residuals indicated that each topic was strongly associated with a distinct source type, suggesting that the model successfully captured meaningful thematic structure.

*Penalized Multinomial Logistic Regression*

To further examine the probability of topic assignment based on source type and the change in probability of topic assignment as source type changes, we used multinomial logistic regression. The complication of this analysis is that our data has separation issue, where the predictor source type largely predicts the topic assignment which leads to extremely large coefficients and standard errors caused by infinite maximum likelihood estimation. We thus used the brmultinom() function in the brglm2 package in R to fit the multinomial logistic regression using bias reduction methods and maximum penalized likelihood estimation with powers of Jeffreys prior as penalty. Supplementary Table 3A reports the estimates in both log odds ratio and odds ratio scales using the predicted topic labels from the baseline ldaMallet model. Supplementary Table 3B reports the equivalent using the predicted topic labels from the SeededLDA/GuidedLDA model.

*Interpretation of Coefficients*

Below is the interpretation of coefficients using the predicted topic labels from the baseline LDAMallet model, which can be followed to examine the interpretation of coefficients (**Supplementary Table 4**) for the semi-supervised model.

- The log odds of being assigned to topic 2 vs. to topic 1 will increase by 0.336 if changing the source type from practice manual to Sanskrit text.

The odds of being assigned to topic 2 relative to topic 1 will increase by (1.400-1) 40% if changing the source type from practice manual to Sanskrit text.

- The log odds of being assigned to topic 2 vs. to topic 1 will decrease by 7.051 if changing the source type from practice manual to scientific research.

The odds of being assigned to topic 2 relative to topic 1 will decrease by (1-0.001) 99.9% if changing the source type from practice manual to scientific research.

- The log odds of being assigned to topic 2 vs. to topic 1 will decrease by 0.651 if changing the source type from practice manual to yoga studies.

The odds of being assigned to topic 2 relative to topic 1 will decrease by (1-0.522) 47.8% if changing the source type from practice manual to yoga studies.

- The log odds of being assigned to topic 3 vs. to topic 1 will not change if changing the source type from practice manual to Sanskrit text.

The odds of being assigned to topic 3 relative to topic 1 will not change if changing the source type from practice manual to Sanskrit text.

- The log odds of being assigned to topic 3 vs. to topic 1 will decrease by 2.608 if changing the source type from practice manual to scientific research.

The odds of being assigned to topic 3 relative to topic 1 will decrease by (1-0.074) 92.6% if changing the source type from practice manual to scientific research.

- The log odds of being assigned to topic 3 vs. to topic 1 will increase by 3.219 if changing the source type from practice manual to yoga studies.

The odds of being assigned to topic 3 relative to topic 1 will increase by (25.000-1) 2400% if changing the source type from practice manual to yoga studies.

- The log odds of being assigned to topic 4 vs. to topic 1 will increase by 4.779 if changing the source type from practice manual to Sanskrit text.

The odds of being assigned to topic 4 relative to topic 1 will increase by (119.000-1) 11800% if changing the source type from practice manual to Sanskrit text.

- The log odds of being assigned to topic 4 vs. to topic 1 will decrease by 4.217 if changing the source type from practice manual to scientific research.

The odds of being assigned to topic 4 relative to topic 1 will decrease by (1- 0.015) 98.5% if changing the source type from practice manual to scientific research.

- The log odds of being assigned to topic 4 vs. to topic 1 will increase by 1.184 if changing the source type from practice manual to yoga studies.

The odds of being assigned to topic 4 relative to topic 1 will increase by (3.267-1) 226.7% if changing the source type from practice manual to yoga studies.

1.3.9 Sensitivity Analysis

The decision to analyze exactly four topics appears to be supported primarily by coherence metrics. Nevertheless, topic modeling can produce different solutions depending on parameter settings. Including a brief sensitivity analysis showing whether similar thematic structures emerge with three, five, or six topics would strengthen confidence in the robustness of the findings.

We tuned the three hyperparameters simultaneously: (1) number of topics; (2) optimize_interval: it optimizes alpha (a prior belief on document-topic distribution) and beta (a prior belief on topic-word distribution) automatically; and (3) iterations: number of training iterations. Below are the values of hyperparameters that reached the highest topic coherence score for two, three, five, and six topics.

num_topics=2, optimize_interval=3000, iterations=6000

num_topics=3, optimize_interval=500, iterations=6000

num_topics=5, optimize_interval=500, iterations=3000

num_topics=6, optimize_interval=3000, iterations=6000

We fitted the LDA Mallet Model with these hyperparameter values and visualized the results using pyLDAvis interactive plot. It showed that similar thematic structures emerged with two, three, five, or six topics and 4-topic model is the best model. First, the pyLDAvis plots kept producing well-separated topics until the number of topics reached 4. When the number of topics reached 5 or more, a few topics began to overlap with each other to form a cluster of topics. For example, when the number of topics was 5, 2 topics overlapped with each other. When the number of topics was 6, two topics overlapped and another two topics overlapped. Overlapping topics implied redundant topics that would need a merge. Although with these overlap, they clearly show the pattern of four well-separated topics (or clusters of topics). 2-topic and 3-topic showed well-separated topics with roughly equal inter-topic distance. For 4-topic model, the topics were still well-separated although some topics were closer to each other. This suggested that there were still four distinctive topics.

Second, pyLDAvis plots showed that 5 or more topics began to have unequal sizes of circles—one larger circle with several smaller overlapping circles and plenty of distance between the larger one and smaller ones. Smaller overlapping topics suggested possible topic redundancy and an overly granular model. Comparing 3-topic versus 4-topic, both produced well-separated circles with equal sizes, but 4-topic produced smaller circles and shorter distance among circles. These are expected because the corpus prevalence is being redistributed across four topics rather than three. And the fact that the four circles remain well separated without overlapping means that the 4-topic model is uncovering a meaningful substructure that the 3-topic model was combining and introducing the fourth topic didn't obviously destroy topic distinctiveness. We saw a transition from an under-specified model (2- or 3-topic) to a reasonable model (4-topic) to an over-specified model (5- or 6-topic), which would make 4-topic the best candidate assuming its topics are substantively interpretable.

Third, we looked at the top probable terms of each topic to examine topic coherence. In 2-topic model, both topics were “undercooked”—meaning that rather than capturing one coherent theme, there were a variety of themes embedded in this topic, all mixed. Topic 1 mixed themes about “state, spiritual, union, power”, about “term, word, time”, and about “call, knowledge, yogin, action”. Topic 2 mixed themes about “health, intervention, mental, stress, effect” and about “techniques, system, ancient”. In 3-topic model, Topic 1 mixed themes about “state, spiritual, union, nature, power” and about “term, word, techniques, system, modern, century”. Topics 2 and 3 had a coherent theme. Here, these models appeared to be grouping multiple distinct topics into a single topic. Accordingly, we ran the model with a greater number of topics, so it had the space to break these topics apart.

In 5- and 6-topic models, we found “overcooked” topics—rather than being too broad or confounded to be interpretable, these topics were too granular. For example, in 5-topic model, top terms in Topics 2 and 5 have moderate-to-strong conceptual overlap. Terms “mind, spiritual, understand, practice” in Topic 2 have strong semantic relationships with terms “consciousness, soul, describe, union” in Topic 5. Terms “call, sense, liberation” appear in both Topics 4 and 5. Similarly, terms “knowledge, yogin, action, brahman, free” in Topic 4 have strong semantic relationships with terms “define, teach, process, perfection, liberation” in Topic 5. In 6-topic, terms “physical, study, include, control” appear in both Topics 1 and 3. Terms “practice, tradition, discipline, religious, patanjali, method, meaning” in Topic 4 have strong semantic relationships with terms “breath, guru, ritual, worship, cessation, method, transcendent/ultimate” in Topic 6. To bring those words together into a single topic, we need to reduce the total number of topics to encourage the model to merge them.
